# Polymeric mechanical amplifiers of immune cytokine-mediated apoptosis

**DOI:** 10.1038/ncomms14179

**Published:** 2017-03-20

**Authors:** Michael J. Mitchell, Jamie Webster, Amanda Chung, Pedro P. G. Guimarães, Omar F. Khan, Robert Langer

**Affiliations:** 1 Department of Chemical Engineering, David H. Koch Institute for Integrative Cancer Research, MIT, Cambridge, Massachusetts 02139, USA

## Abstract

Physical forces affect tumour growth, progression and metastasis. Here, we develop polymeric mechanical amplifiers that exploit *in vitro* and *in vivo* physical forces to increase immune cytokine-mediated tumour cell apoptosis. Mechanical amplifiers, consisting of biodegradable polymeric particles tethered to the tumour cell surface via polyethylene glycol linkers, increase the apoptotic effect of an immune cytokine on tumour cells under fluid shear exposure by as much as 50% compared with treatment under static conditions. We show that targeted polymeric particles delivered to tumour cells *in vivo* amplify the apoptotic effect of a subsequent treatment of immune cytokine, reduce circulating tumour cells in blood and overall tumour cell burden by over 90% and reduce solid tumour growth in combination with the antioxidant resveratrol. The work introduces a potentially new application for a broad range of micro- and nanoparticles to maximize receptor-mediated signalling and function in the presence of physical forces.

## Abstract (web summary)

Fluid shear stress plays a critical role in receptor-mediated signalling and has been shown to sensitize cancer cells to apoptosis. Here, Mitchell *et al*. introduce polymer micro- and nanoparticles tethered to tumour cells to amplify fluid shear stress effects, and find that they can enhance immune cytokine-mediated apoptosis of tumour cells *in vitro* and *in vivo*.

Receptor-mediated signalling in biological systems is essential for the exchange of information between cells and the extracellular environment, and contributes to important cellular phenomenon including growth, survival, differentiation, ageing and death^1^. To exert greater control of receptor–ligand interactions and signalling, nanotechnology-based platforms that interface with the cell surface are being developed^2^. Nanoscale surface engineering of materials is being used to mimic receptor–ligand interactions *in vivo*, to support cell survival and guide cellular behaviour for various biomedical applications^3^. In addition, nanoparticles have shown potential in manipulating receptor-mediated signalling, exerting control over receptor clustering for the induction of receptor-mediated signalling^4^,^5^. Nanoparticles have also demonstrated promise in the spatiotemporal control of gene interference within cells that can be remotely controlled using light^6^. The majority of studies have demonstrated control of receptor-mediated phenomena using nanoparticles *in vitro*, and few studies have demonstrated such control *in vivo*.

Receptor-mediated signalling in response to mechanical forces is also under intense study, and plays an important role in embryonic development and adult physiology, while also contributing to numerous diseases including atherosclerosis, osteoporosis and cancer^7^. For example, blood flow exerts shear stress on endothelial cell-lined vessels *in vivo*, and plays an essential role in vascular remodelling and branching^8^. Conversely, atherosclerotic plaques that can trigger cardiovascular disease preferentially form under disturbed physiological flows and shear stress at the branch points of vessels^9^. Malignant cells are also exposed to a wide range of physiological fluid shear forces *in vivo*, including high shear stress (0.5–30.0 dyn cm^−2^) in the circulation generated by blood flow and low shear stress (∼0.1 dyn cm^−2^) in soft tissues generated by interstitial flow^10^. Such forces can trigger mechanotransduction in tumour cells at the receptor level both *in vitro* and *in vivo*, which can affect tumour progression^11^,^12^,^13^,^14^,^15^.

Drug resistance also plays a key role in tumour progression, and is commonly implicated in the failure of therapeutic regimens. Despite an initial therapeutic response, tumours, in part because of their heterogeneity, can regrow and manifest resistance to the initially successful therapeutic^16^,^17^. Thus, many drugs are used in combination with other chemical agents to sensitize tumour cells and overcome drug resistance. Therapies based on the tumour necrosis factor-related apoptosis-inducing ligand (TRAIL), an immune cytokine, are of heightened interest because of its capacity to trigger receptor-mediated apoptosis in cancer cells. TRAIL exerts cytotoxic effects on tumour cells while sparing normal cells, and has shown promise in treating solid tumours and metastatic tumour cells^18^,^19^. As such, TRAIL has long been a target for clinicians and researchers as a cancer therapeutic that avoids the debilitating effects of conventional chemotherapies^20^. Several drugs targeting TRAIL death receptors 4 (DR4) and 5 (DR5) on the tumour cell surface are currently undergoing clinical trials, and show little toxicity^21^. Although treatment has shown some efficacy in subsets of patients, TRAIL resistance remains a major clinical barrier and limits broad efficacy^21^.

To overcome resistance, several agents have been combined with TRAIL to sensitize tumour cells to apoptosis, including conventional chemotherapeutics (that is, doxorubicin, cisplatin), proteasome inhibitors, quercetin, BCL2 (B-cell lymphoma 2) inhibitors, IAP (inhibitor of apoptosis protein) antagonists, CD20 antibodies, irradiation, Sorafenib, aspirin, histone deacetylase inhibitors and natural products such as resveratrol and piperlongumine^22^. Although numerous chemical sensitization approaches have been demonstrated, it is difficult to assess the appropriate sensitizer for a particular patient, given that multiple known TRAIL resistance mechanisms exist, in addition to those that have not been identified^23^. Furthermore, several chemical sensitizers, such as doxorubicin, induce systemic toxicity *in vivo* and thus negate the low toxicity advantages of TRAIL administration^22^. Although numerous groups have studied the effect of chemical sensitizers, to our knowledge no one has explored leveraging mechanical stimulation as a means to increase TRAIL sensitivity while sparing normal cells *in vivo*.

Herein, we develop polymeric mechanical amplifiers that leverage fluid shear stress to enhance receptor-mediated tumour cell apoptosis. The approach, consisting of degradable and nondegradable polymeric particles bound to the cell surface via polyethylene glycol (PEG) linkers, enhance receptor-mediated signalling and apoptosis of TRAIL-treated tumour cells in the presence of fluid shear forces. When administered *in vivo*, surface-bound particles enhance the apoptotic effect of a subsequent treatment of soluble TRAIL, and reduce circulating tumour cell burden and solid tumour growth. These mechanical amplifiers have negligible toxic effects on normal cells, and can be combined with a range of natural products to exert synergistic apoptotic effects on tumour cells.

## Results

### Fluid shear increases immune cytokine-mediated apoptosis

To examine how physical forces affect TRAIL-mediated tumour cell apoptosis, tumour cells in suspension were treated with TRAIL *in vitro* and exposed to physiologically relevant fluid shear stress (Supplementary Fig. 1a). In the presence of fluid shear stress, significant increases in tumour cell killing were observed in TRAIL-treated human colon, prostate and breast tumour cells as compared with those treated under static conditions (Supplementary Fig. 1b–d). Increased tumour cell killing in the presence of fluid forces was observed in both TRAIL-sensitive (COLO 205) and TRAIL-resistant (MCF7) tumour cells (Supplementary Fig. 1b–d). Normal cells with negligible TRAIL sensitivity, including human peripheral blood mononuclear leukocytes and human endothelial cell monolayers, were not sensitized to TRAIL-mediated killing upon shear stress exposure (Supplementary Fig. 1h,i). Across a range of fluid shear forces characteristic of those in soft tissues and in the vascular microenvironment, it was evident that increased shear force enhanced TRAIL-mediated tumour cell killing (Supplementary Fig. 1e–g). We then assessed whether shear force exposure increased TRAIL-mediated apoptosis via caspase-mediated signalling, which is triggered upon TRAIL binding to death receptors DR4 and DR5 (ref. 24). Indeed, treatment with the general caspase inhibitor Z-VAD-FMK abolished TRAIL-mediated tumour cell killing in the presence of fluid shear stress (Supplementary Fig. 1j). These data suggest that physiological forces exerted on tumour cells enhance the therapeutic effect of TRAIL. Building upon previous work, which suggested that shear forces increase the killing of TRAIL-sensitive tumour cells *in vitro*^25^, this work indicates that mechanical forces increased both TRAIL-sensitive and TRAIL-resistant tumour cell killing, with negligible toxic effects on normal cells.

### Polymeric mechanical amplifiers

**Figure 1:**
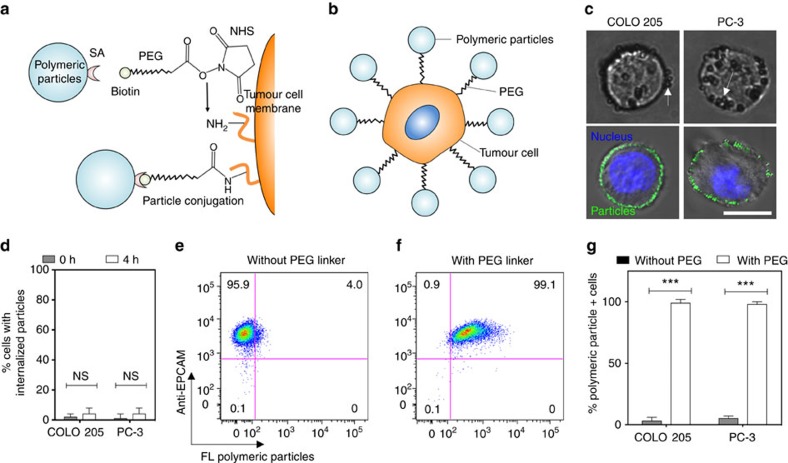
Functionalization of the tumour cell surface with polymeric particles. (**a**,**b**) NHS–PEG–biotin linkers (**a**) were used to conjugate a range of streptavidin-functionalized polymeric particles to the tumour cell surface (**b**). (**c**) Brightfield (top) and confocal (bottom) micrographs of polymeric polystyrene (PS) particles conjugated to the surface of colon (COLO 205; left) and prostate (PC-3; right) tumour cell lines. The 500 nm diameter PS particles bound to tumour cells in brightfield micrographs. Brightfield micrographs show 500 nm diameter PS particles bound to tumour cells. The 200 nm diameter PS particles bound to tumour cells in confocal micrographs. Confocal micrographs shown 200 nm diameter PS particles bound to tumour cells. Green indicates polymeric PS particles and blue indicates nucleus. Scale bar, 10 μm. (**d**) Percentage of tumour cells with internalized fluorescent PS particles, immediately after and 4 h post functionalization. Internalized fluorescent PS particles quantified using a Trypan blue fluorescence quenching assay and confocal microscopy. *N*=5 per treatment. (**e**,**f**) Flow cytometry plots of epithelial cell adhesion molecule (EpCAM)+ tumour cells functionalized with fluorescent PS particles in the absence (**e**) and presence (**f**) of a PEG linker. *N*=5 per treatment. (**g**) Percentage of fluorescent PS particle+ tumour cells after functionalization with a PEG linker. Data are reported as the mean±s.e. Different treatment groups were compared for statistical significance using Student's two-tailed *t*-test. *N*=5 per treatment. NHS, *N*-hydroxysuccinimide; NS, not significant; SA, streptavidin. ***P<0.001.

Although our data suggest that mechanical forces sensitize tumour cells to receptor-mediated apoptosis, fluid shear forces are highly variable *in vivo*. Thus, we investigated whether biocompatible polymeric particles conjugated to the tumour cell surface act to locally amplify the forces exerted on the tumour cell membrane. By locally increasing the physical force exerted on the cell surface, it is possible to amplify the apoptotic effect of TRAIL on target tumour cells without altering the fluid forces of the surrounding area. To accomplish this, we stably conjugated polymeric particles across a range of sizes (diameter: 100–1,000 nm) to the tumour cell surface via free amine coupling using *N*-hydroxysuccinimide (NHS)–PEG_12_–biotin heterobifunctional linkers (Fig. 1a,b). For this study, we chose nondegradable polystyrene (PS) and degradable poly(lactic-co-glycolic acid) (PLGA) particles given their biocompatibility, biodegradability and current use in *in vivo* clinical applications^26^,^27^,^28^. Particles were stably bound to the surface of colon and prostate tumour cells (Fig. 1c), with minimal internalization observed in the overall cell population after treatment and 4 h post treatment (Fig. 1d). Although some polymeric particles adsorbed to the tumour cell surface without PEG linkers in a nonspecific manner, these particles were easily removed from ∼95% of the overall cell population during mild cell washing steps (Fig. 1e,g). However, polymeric particles conjugated to the cell surface using PEG linkers remained bound to >99% of the overall cell population after exposure to identical washing steps (Fig. 1f,g). Hundreds of polymeric particles were stably conjugated to the tumour cell surface using this technique, with negligible effects on cell viability (Supplementary Fig. 3). Fluorescence readings indicated that a negligible amount of fluorescent particles remained in suspension after functionalization as compared with controls (Supplementary Fig. 4a). In addition, flow cytometry results showed a normal Gaussian distribution of fluorescent cells post functionalization, indicating that the majority of the tumour cell population was uniformly functionalized with particles (Supplementary Fig. 4b). Furthermore, conjugation of particles to the tumour cell surface did not significantly interfere with the ability of TRAIL to interact with death receptors DR4 and DR5, as no significant differences in cell viability were observed post treatment (Supplementary Fig. 5). This finding suggests that PEG linkers enable stable conjugation of polymeric particles to the tumour cell surface with minimal internalization, and has negligible effects on both cell viability and TRAIL-mediated signalling under static conditions.

### Mechanical amplification of tumour cell apoptosis *in vitro*

**Figure 2:**
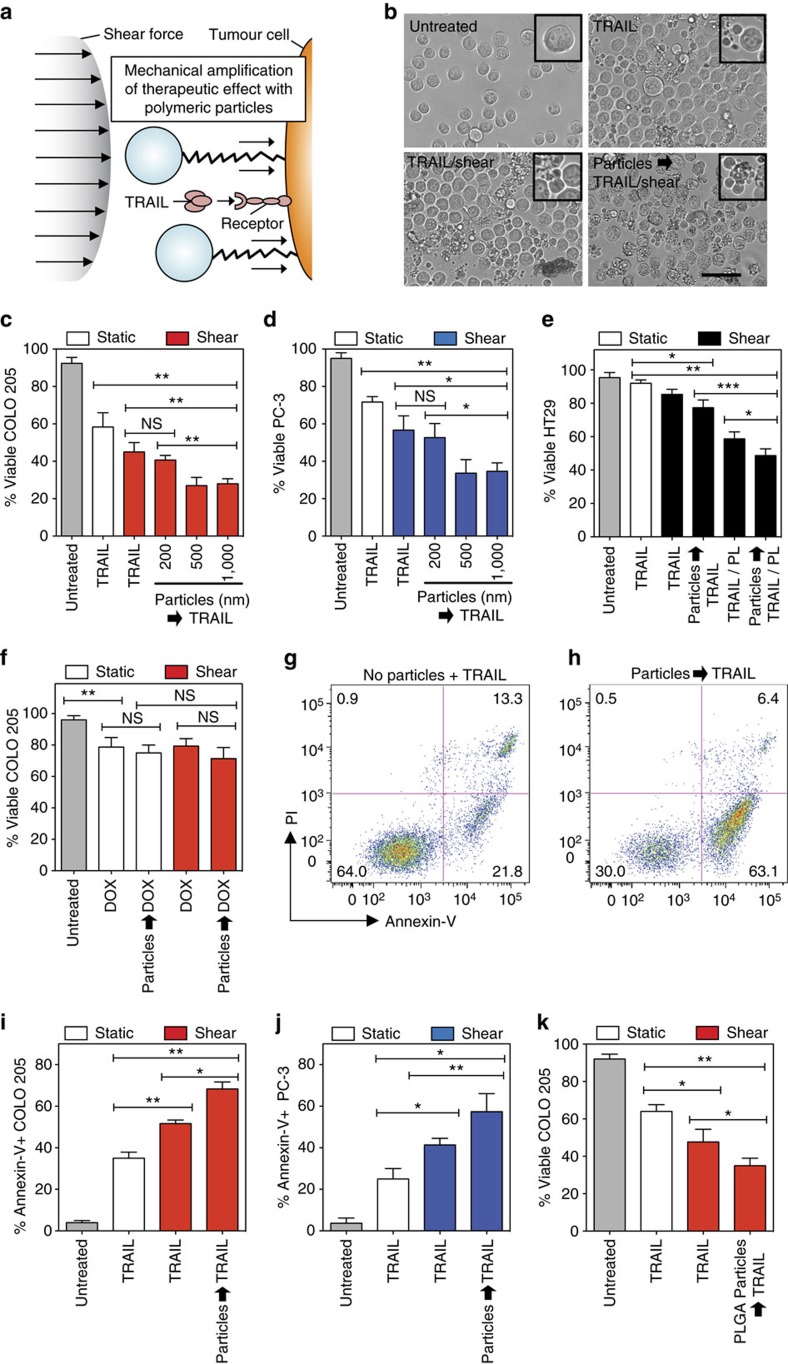
Polymeric particles conjugated to tumour cell surface amplify TRAIL-mediated apoptosis in the presence of fluid shear stress. (**a**) Schematic of polymeric particles acting as mechanical amplifiers by increasing TRAIL-mediated tumour cell apoptosis in presence of a fluid shear force. (**b**) Brightfield micrographs of particle-functionalized COLO 205 tumour cells after 30 min of exposure to various treatment conditions. TRAIL-treated samples incubated with 0.1 μg ml^−1^ TRAIL for 30 min. Sheared samples were exposed to a fluid shear stress of 4.0 dyn cm^−2^. Tumour cells were treated with 240 PS (500 nm diameter) particles per tumour cell before exposure to TRAIL and fluid shear stress. Insets denote viable and apoptotic tumour cells after treatment conditions. Scale bar, 30 μm. (**c**,**d**) Viability of particle-functionalized COLO 205 (**c**) and PC-3 (**d**) tumour cells after treatment with TRAIL (0.1 μg ml^−1^) in the presence of fluid shear stress. *N*=4 per treatment. (**e**) Viability of particle-functionalized, TRAIL-resistant HT29 tumour cells after treatment with TRAIL (0.1 μg ml^−1^) in the presence of fluid shear stress for 6 h. For combination therapies, cells were treated with 15 μM piperlongumine (PL) in addition to TRAIL (0.1 μg ml^−1^). *N*=4 per treatment. (**f**) Viability of particle-functionalized COLO 205 tumour cells after treatment with doxorubicin (DOX; concentration: 20 μM) in the presence of fluid shear stress. *N*=4 per treatment. Particle diameter: 500 nm. (**g**,**h**) Annexin-V/propidium iodide (PI) flow cytometry plots of nonfunctionalized (**g**) and particle (500 nm)-functionalized (**h**) PC-3 tumour cells after treatment with TRAIL (0.1 μg ml^−1^) in the presence of static conditions and fluid shear stress, respectively. Particle diameter: 500 nm. *N*=5 per treatment. (**i**,**j**) Percentage of annexin-V+, particle-functionalized COLO 205 (**i**) and PC-3 (**j**) tumour cells after treatment with TRAIL (0.1 μg ml^−1^) in the presence of fluid shear stress. *N*=5 per treatment. (**k**) Viability of biodegradable PLGA particle-functionalized COLO 205 tumour cells after treatment with TRAIL (0.1 μg ml^−1^) in the presence of fluid shear stress. Data are reported as the mean±s.e. Different treatment groups were compared for statistical significance using Student's two-tailed *t*-test. Particle diameter: 500 nm. *N*=5 per treatment. *P<0.05, **P<0.01 and ***P<0.001. NS, not significant.

Given that fluid shear stress enhanced TRAIL-mediated tumour cell killing (Supplementary Fig. 1), we then investigated whether polymeric particles conjugated to the tumour cell surface amplify the apoptotic effect of TRAIL in the presence of shear forces (Fig. 2a). Under both shear and static conditions, particle conjugation to the tumour cell surface had no effect on viability in the absence of TRAIL (Supplementary Fig. 6). Furthermore, conjugation of particles across a range of sizes (100–1,000 nm) to the cell surface had no effect on TRAIL-mediated killing under static conditions (Supplementary Fig. 7). However, particles tethered to the cell surface amplified TRAIL-mediated colon and prostate cancer cell killing in the presence of fluid shear stress (Fig. 2b–d). Specifically, conjugation of particles of increased size had a pronounced effect on TRAIL-mediated tumour cell killing in the presence of fluid shear stress as compared with treatment in the absence of particles. Increased tumour cell killing in the presence of larger particles could be due to greater compressive forces exerted on the tumour cell membrane in the presence of fluid shear stress. Two spherical particles will experience a compressive force (*F*_c_) between them when colliding in a linear shear flow that scales as *F*_c_∼*μGab*, where *μ* is the fluid viscosity, *G* is the shear rate, and *a* and *b* are the radii of the smaller and larger sphere, respectively^29^. Therefore, a 10 μm diameter tumour cell colliding with a 100 nm particle will experience 10 times the compressive force of a 10 nm particle colliding with a tumour cell. Similar effects were not observed in normal cells, as particle conjugation to both human peripheral blood mononuclear cells and human endothelial cell monolayers had no significant effect on TRAIL-mediated killing (Supplementary Fig. 8). This finding suggests that polymeric particles can be utilized to amplify TRAIL-mediated tumour cell killing in the presence of fluid shear stress while sparing normal cells.

TRAIL resistance in tumour cell types is a major challenge to its broad use in cancer therapy^22^,^30^. To examine the potential of our approach to overcome resistance, we conducted experiments using HT29 colon cancer cells that have been shown to be TRAIL resistant^31^,^32^,^33^,^34^. Our results confirm that TRAIL treatment under static conditions has minimal effects on HT29 cell viability (Fig. 2e). However, a significant decrease in HT29 cell viability was measured when cells were functionalized with particles and then treated with TRAIL in the presence of fluid shear stress (Fig. 2e). We then assessed whether our particle-based approach in combination with piperlongumine, a natural alkaloid shown to sensitize tumour cells to TRAIL via upregulation of DR5 expression, can further increase tumour cell killing^35^. Indeed, combining piperlongumine with our approach induced a fivefold greater tumour cell death than TRAIL treatment alone. These results suggest that our particle-based approach can act in combination with chemical sensitizers to increase TRAIL-resistant tumour cell killing in the presence of physical forces. Given that TRAIL combination therapies with various classes of sensitizers are being explored in preclinical and clinical trials^22^,^30^,^36^, we envision that our nontoxic, particle-based approach can act in combination with sensitizers to exert synergistic apoptotic effects on TRAIL-resistant tumour cells.

We then compared mechanical amplification of tumour cell killing with treatment with doxorubicin. Doxorubicin also triggers tumour cell apoptosis, but through intracellular mechanisms including inhibition of topo-isomerase II and DNA intercalation^37^. Although polymeric particles amplified the effect of TRAIL in the presence of fluid shear stress (Fig. 2c–e), they did not enhance the therapeutic effect of doxorubicin (Fig. 2f), demonstrating that such an approach is specific to receptor-mediated therapeutics. Upon analysing the mode of tumour cell death using an annexin-V/propidium iodide (PI) assay, increased tumour cell killing using our approach occurred almost entirely via increased apoptosis (Fig. 2g–j). Conjugation of particles to the cell surface nearly doubled the number of apoptotic prostate and colon cancer cells in the presence of fluid shear stress as compared with treatment under static conditions (Fig. 2i,j). Furthermore, no significant differences in tumour cell necrosis were observed (Supplementary Fig. 9). Given that TRAIL primarily triggers cell death via apoptosis rather than necrosis, the annexin-V/PI assay further suggests that mechanical amplification of apoptosis using polymeric particles is TRAIL specific and does not exert separate cytotoxic effects.

In addition to nondegradable particles, we sought to utilize biodegradable polymeric PLGA particles to mechanically amplify TRAIL-mediated apoptosis. Biodegradable particles such as PLGA are particularly advantageous for *in vivo* administration, where the material can degrade in a safe manner^38^,^39^. PLGA particles functionalized with an epithelial cell adhesion molecule (EpCAM)-targeting antibody, which binds to EpCAM expressed on tumour cells of epithelial origin^40^, were bound to the tumour cell surface with minimal effects on cell viability under shear and static conditions (Supplementary Fig. 10a). Furthermore, PLGA particles did not hinder TRAIL-mediated killing under static conditions (Supplementary Fig. 10b). Similar to nondegradable particles (Fig. 2b–e), biodegradable particles amplified the apoptotic effect of TRAIL in the presence of fluid shear stress as compared with samples in the absence of particles (Fig. 2k). This finding suggests that biodegradable polymeric particles can also be utilized to amplify TRAIL-mediated apoptosis in the presence of fluid shear stress.

**Figure 3:**
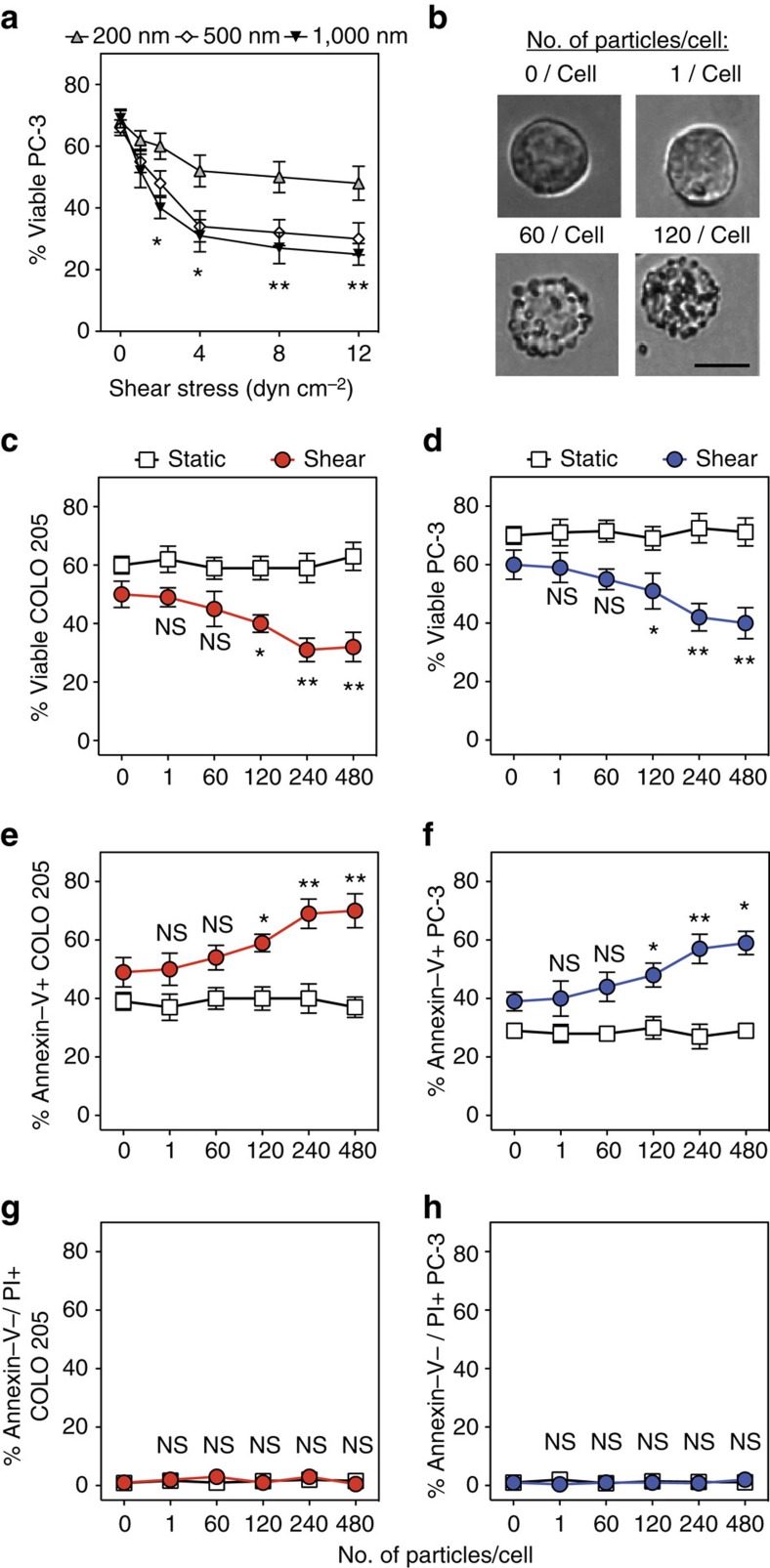
Increased shear stress, particle diameter and number of particles conjugated to tumour cell surface enhance TRAIL-mediated apoptosis. (**a**) Viability of particle-functionalized PC-3 tumour cells treated with TRAIL in the presence of a range of shear forces (1.0–12.0 dyn cm^−2^) across a range of particle sizes (diameter: 200–1,000 nm). (**b**) COLO 205 tumour cells treated with 0–120 PS particles per cell in suspension. Scale bar, 10 μm. (**c**,**d**) Viability of particle-functionalized COLO 205 (**c**) and PC-3 tumour cells (**d**) treated with TRAIL in the presence of fluid shear stress. *N*=5 per treatment. (**e**,**f**) Percentage of annexin-V+ particle-functionalized COLO 205 (**e**) and PC-3 cells (**f**) treated with TRAIL in the presence of fluid shear stress. *N*=5 per treatment. (**g**,**h**) Percentage of annexin-V-/PI+ particle-functionalized COLO 205 (**g**) and PC-3 cells (**h**) treated with TRAIL in the presence of fluid shear stress (shear stress: 4.0 dyn cm^−2^). All tumour cells were incubated with 0–480 PS particles (500 nm diameter) per cell before all treatments. *N*=5 per treatment. TRAIL concentration: 0.1 μg ml^−1^ for all TRAIL-treated samples. Shear stress: 4.0 dyn cm^−2^ for all samples exposed to shear. Data are reported as mean±s.e. Different treatment groups were compared for statistical significance using Student's two-tailed *t*-test. NS, not significant. *P<0.05 and **P<0.01.

Our results with larger polymeric particles conjugated to the tumour cell surface showed increased TRAIL-mediated tumour cell killing under shear, suggesting that greater force exerted by larger particles increased the effect of TRAIL (Fig. 2). To assess the effects of both shear stress exposure and particle size, tumour cells were conjugated with polymeric particles across a range of sizes (200–1,000 nm) before TRAIL treatment under varied fluid shear stress (1.0–12.0 dyn cm^−2^; Fig. 3a). Higher shear stress values were not examined *in vitro* because of cell delamination and reduced cell recovery from the viscometer device (Supplementary Fig. 11). Particles across the range of sizes increased TRAIL-mediated tumour cell killing with increasing fluid shear stress exposure (Fig. 3a). In addition, increased TRAIL-mediated tumour cell killing was observed when cells were bound to larger particles at a given fluid shear stress as compared with smaller particles (Fig. 3a). For example, although a 200 nm particle had minimal effect on TRAIL-mediated tumour cell killing at a low fluid shear stress (1.0 dyn cm^−2^), functionalization with larger particles (1,000 nm) significantly increased tumour cell killing (Fig. 3a). These results suggest that particle size and shear stress exposure both act to modulate TRAIL-mediated tumour cell killing. In addition, we treated tumour cells with varying numbers of particles (concentration: 0–480 particles per cell) before TRAIL treatment under shear exposure (Fig. 3b). In the absence of TRAIL, increasing numbers of particles had no significant effect on cell viability under shear or static conditions (Supplementary Fig. 12). In the presence of TRAIL, increased particle conjugation increased tumour cell killing under shear conditions (Fig. 3c,d). Increased particle conjugation did not affect tumour cell killing under static conditions (Fig. 3c,d), providing further evidence that the apoptotic effect of TRAIL is amplified by particles specifically in the presence of fluid shear stress. Furthermore, the apoptotic effect of TRAIL increased with greater numbers of particles conjugated to the cell surface (Fig. 3e,f), whereas no significant differences in cellular necrosis were measured (Fig. 3g,h). In addition, particle functionalization had no effect on tumour cell apoptosis and necrosis in the absence of TRAIL, regardless of the number of particles (Supplementary Fig. 13). These data provide further evidence that mechanical amplification of TRAIL-mediated tumour cell killing occurs via apoptosis, and can be modulated by (1) altering particle size, (2) increasing shear force exposure and (3) increasing the number of particles tethered to the tumour cell surface.

### Mechanical amplification of caspase signalling

**Figure 4:**
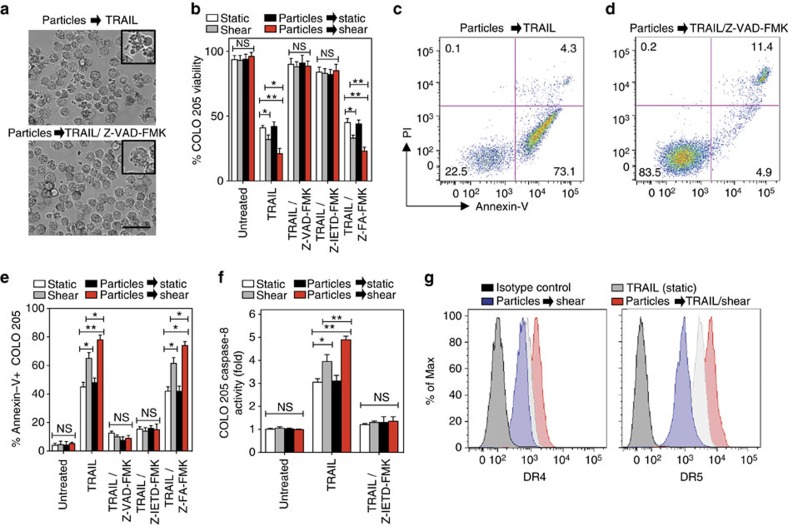
Amplification of TRAIL apoptotic effect via polymeric particles conjugation is caspase dependent and increases death receptor expression. (**a**) Brightfield micrographs of particle (diameter: 500 nm)-functionalized COLO 205 tumour cells treated with 0.1 μg ml^−1^ TRAIL under fluid shear stress exposure (shear stress: 4.0 dyn cm^−2^) for 1 h in the absence and presence of 50 μM pan caspase inhibitor Z-VAD-FMK. (**b**) Viability of particle-functionalized COLO 205 tumour cells treated with 0.1 μg ml^−1^ TRAIL under fluid shear stress exposure for 1 h in the presence of 50 μM pan caspase inhibitor Z-VAD-FMK, 50 μM caspase-8 inhibitor Z-IETD-FMK or 50 μM caspase negative control inhibitor Z-FA-FMK. *N*=4 for all treatments. (**c**,**d**) Annexin-V/propidium iodide (PI) flow cytometry plots of particle-functionalized COLO 205 tumour cells treated with 0.1 μg ml^−1^ TRAIL in the presence of a fluid shear stress for 1 h without and with Z-VAD-FMK treatment. *N*=4 for all treatments. (**e**) Annexin-V quantification of particle-functionalized COLO 205 tumour cells treated with 0.1 μg ml^−1^ TRAIL under fluid shear stress exposure for 1 h in the presence of 50 μM pan caspase inhibitor Z-VAD-FMK, 50 μM caspase-8 inhibitor Z-IETD-FMK or 50 μM caspase negative control inhibitor Z-FA-FMK. *N*=4 for all treatments. (**f**) Caspase-8 activity of particle-functionalized COLO 205 tumour cells after treatment with TRAIL (0.1 μg ml^−1^) in the presence and absence of fluid shear stress for 1 h. *N*=4 for all treatments. (**g**) TRAIL death receptor (DR) 4 and 5 expression after treatment of particle-functionalized COLO 205 tumour cells with TRAIL (0.1 μg ml^−1^) in the presence and absence of fluid shear stress for 1 h. *N*=5 for all treatments. Data are reported as mean±s.e. Different treatment groups were compared for statistical significance using Student's two-tailed *t*-test for two conditions and one-way analysis of variance (ANOVA) for multiple comparisons. Percent of max represents the number of events normalized according to FlowJo algorithms. *P<0.05 and **P<0.01. NS, not significant.

To assess whether amplification of receptor-mediated apoptosis in the presence of fluid shear stress is dependent on caspase signalling, we treated particle-functionalized tumour cells with the general caspase inhibitor Z-VAD-FMK. Minimal membrane blebbing, indicative of reduced TRAIL-mediated apoptosis, was observed in samples treated with Z-VAD-FMK (Fig. 4a). No significant decreases in viability were measured in Z-VAD-FMK-treated samples as compared with viable controls (Fig. 4b). Given that TRAIL primarily induces extrinsic apoptosis via caspase-8, cells were also treated with the caspase-8 inhibitor Z-IETD-FMK, and similar results were observed compared with general caspase inhibition (Fig. 4b). Furthermore, Annexin-V/PI assays showed reduced apoptosis when treated with Z-VAD-FMK or Z-IETD-FMK (Fig. 4c–e). We then labelled for biomarkers of caspase-8 activation using a caspase-8 activity assay. TRAIL treatment alone induced a 3-fold increase in caspase-8 activity, while TRAIL treatment of particle-functionalized cells induced over a 4.5-fold increase in caspase-8 activity in the presence of fluid shear stress as compared with controls (Fig. 4f). These results suggest that polymeric particles amplify TRAIL-mediated apoptosis in the presence of shear forces via increased caspase-8 activity.

To assess whether mechanical amplification of TRAIL-mediated apoptosis alters death receptor (DR4, DR5) expression on the cell surface, we measured receptor expression of particle-functionalized tumour cells after exposure to TRAIL and fluid shear stress via flow cytometry. TRAIL binding to DR4 and DR5 on the tumour cell surface engages the caspase signalling cascade and triggers apoptosis^41^. In addition, TRAIL treatment alone increases death receptor expression in malignant epithelial cells in a process mediated by nuclear factor-κB activation^42^. Therefore, increased DR4 and DR5 expression on particle-functionalized tumour cells in the presence of shear could explain increased TRAIL-mediated apoptosis due of increased receptor–ligand interactions and subsequent apoptotic signalling. Indeed, flow cytometry analysis showed increased death receptor expression on TRAIL-treated, particle-functionalized tumour cells in the presence of fluid shear stress (Fig. 4g). Shear stress exposure in the absence of TRAIL did not have an effect on death receptor expression, consistent with previous work^25^. Thus, our data suggest that mechanical amplification of TRAIL-mediated apoptosis using polymeric particles is dependent on caspase signalling and could be due to increased death receptor expression. The enhanced apoptotic effect of TRAIL could be because of increased compressive forces exerted by particles conjugated to the tumour cell surface in the presence of fluid shear stress. Such forces act to flatten the biologically inert glycocalyx expressed on the surface of cells, and the physics of force-induced flattening and penetration of cell glycocalyx have been shown to facilitate receptor–ligand interactions^43^,^44^. Given that the glycocalyx is overexpressed on many tumour cell types, increased compressive forces and glycocalyx flattening can potentially increase TRAIL–death receptor interactions, thus increasing both death receptor expression and apoptosis.

### Amplification of tumour cell death *in vivo*

**Figure 5:**
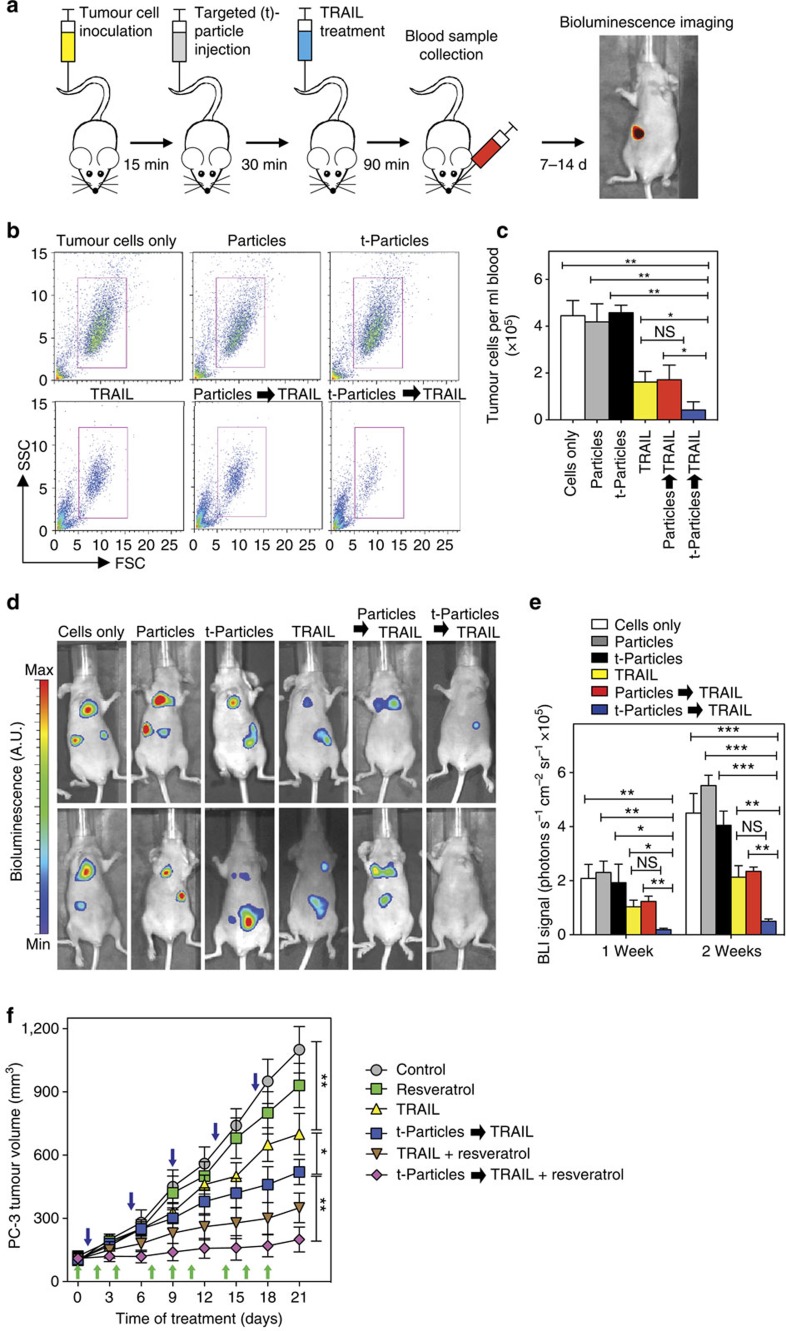
Polymeric particles targeted to tumor cell surface amplify immune cytokinemediated apoptosis *in vivo*. (**a**) Schematic of epithelial cell adhesion molecule (EpCAM)-targeted particle delivery to COLO 205 tumour cells in nude (nu/nu) mice *in vivo*, followed by treatment with TRAIL. Mice were inoculated with COLO 205 tumour cells via tail vein injection (2 × 10^6^ cells), followed by injection of nontargeted and EpCAM-targeted PLGA particles (500 nm diameter; ∼500 particles per tumour cell) 15 min post tumour cell injection. At 30 min post particle injection, mice were treated with TRAIL (0.1 μg ml^−1^ plasma concentration). Tumour cells in blood were collected via submandibular bleed 90 min post TRAIL injection. Tumour cells were detected *in vivo* via whole-body bioluminescent imaging (BLI) at 7 and 14 days post injection. (**b**) Representative flow cytometry plots of GFP+ COLO 205 tumour cells removed after delivery of nontargeted particles (Particles) and EpCAM-targeted particles (t-Particles) followed by TRAIL. FSC, forward scatter; SSC, side scatter. (**c**) Number of viable GFP+ COLO 205 tumour cells per ml mouse blood 90 min post TRAIL treatment of tumour cells *in vivo* under various conditions. Cells only denotes mice treated with tumour cells followed by PBS via tail vein injection. *N*=5 mice for all treatments. (**d**) Representative whole-body BLI images of COLO 205 tumour cells in mice 7 days post injection of particles and targeted particles followed by TRAIL. (**e**) COLO 205 BLI signals in mice 7 and 14 days post injection of COLO 205 tumour cells under various conditions. *N*=5 mice for all treatments. (**f**) PC-3 tumour growth curves after intravenous injections of targeted particles (40 mg kg^−1^) followed by TRAIL (15 mg kg^−1^) 3 h post particle injection. For combination therapies, tumour-bearing nu/nu mice were also treated with the TRAIL-sensitizer resveratrol (30 mg kg^−1^). After tumour formation (100 mm^3^), mice began treatment regimen and tumour volume was measured every 3 days. Blue arrows indicate days where mice were treated with targeted particles, followed 3 h later by TRAIL treatment. Green arrows indicate days where mice were treated with resveratrol via oral gavage. *N*=5 mice for all treatments. Data are reported as the mean±s.e. Different treatment groups were compared for statistical significance using Student's two-tailed *t*-test for two conditions and one-way analysis of variance (ANOVA) for multiple comparisons. *P<0.05, **P<0.01 and ***P<0.001. NS, not significant.

We then assessed whether a targeted particle formulation can amplify TRAIL-mediated apoptosis of tumour cells in the bloodstream *in vivo*, where physical force exposure is highly variable. GFP- and luciferase-expressing COLO 205 tumour cells were injected systemically into the tail vein of nu/nu mice, followed 15 min later by an injection of EpCAM-targeted, PEG-functionalized PLGA particles (Fig. 5a). Given that EpCAM is expressed on tumour cells of epithelial origin with negligible expression on blood cells in the vascular microenvironment, this particle platform enables targeting of EpCAM+ tumour cells in the vasculature. At 30 min post particle treatment, mice were treated with soluble TRAIL via systemic administration (Fig. 5a), enabling the therapeutic to interact with particle-functionalized tumour cells under *in vivo* fluid shear stress exposure. Viable tumour cells in the bloodstream were measured by collecting 200 μl blood samples from mice 90 min post injection via submandibular bleeding (Fig. 5a). Flow cytometry analysis showed a significant decrease in the number of targeted particle-bound tumour cells subsequently treated with soluble TRAIL in the circulation as compared with control mice (Fig. 5b). Using flow cytometry, we measured ∼40,000 tumour cells per ml of blood for mice injected with tumour cells alone, and mice injected with tumour cells followed by targeted or non-targeted particle administration (Fig. 5c). We measured ∼14,000 tumour cells per ml of blood for mice injected with soluble TRAIL as compared with <3,000 tumour cells per ml for mice injected with EpCAM-targeted particles followed by treatment with soluble TRAIL (Fig. 5c). Upon injection with nontargeted particles, we measured similar levels of tumour cells in blood compared with tumour cells treated with soluble TRAIL alone, indicating that EpCAM targeting enables particle binding to the tumour cell surface and is necessary for amplifying the apoptotic effect of TRAIL (Fig. 5c). In addition to reduced tumour cells in the circulation, annexin-V analysis showed that ∼65% of the particle-bound tumour cell population in circulation was apoptotic after TRAIL treatment, whereas ∼45% of the tumour cell population treated with TRAIL in the absence of particles were apoptotic (Supplementary Fig. 14). Given that tumour cells administered via tail vein injection can lodge within the vasculature >2 h post injection^45^, we utilized whole-body bioluminescence imaging (BLI) to track the remaining tumour cell burden 7 and 14 days post injection (Fig. 5a). Tumour cell burden was readily apparent within control nu/nu mice treated with tumour cells 7 days post injection, along with tumour cells treated with targeted or nontargeted particles in the absence of TRAIL treatment (Fig. 5d). Administration of soluble TRAIL alone reduced the number of tumour cells *in vivo*, as measured via BLI imaging (Fig. 5d). The apoptotic effect of soluble TRAIL on tumour cells was significantly increased *in vivo* after pretreatment with EpCAM-targeted particles, as BLI signals were significantly reduced 7 days post injection. Quantitative analysis showed a >90% reduction in BLI signal for tumour cells treated with EpCAM-targeted particles followed by soluble TRAIL administration *in vivo* as compared with controls (Fig. 5e).

In addition to tumour cells in the circulation, we assessed whether our approach could amplify TRAIL-mediated apoptosis in solid tumour models, where tumour cells are exposed to a variety of physical forces including blood flow from leaky tumour vasculature, interstitial flows between blood and lymphatic circulation as well as interstitial fluid pressures generated within solid tumours^10^,^46^,^47^,^48^. Using a prostate cancer, EpCAM+ PC-3 xenograft model, we first confirmed that EpCAM-targeted PLGA particles localized within the tumour 3 h post injection using *in vivo* fluorescence imaging (Supplementary Fig. 15a). Harvesting of mouse organs showed that particles mainly accumulated within the tumour and liver, with some particle clearance to the kidneys (Supplementary Fig. 15b). Over a 21-day treatment period, we measured a significant decrease in PC-3 tumour growth in mice treated with targeted particles followed by TRAIL administration (3 h post particle injection), compared with TRAIL treatment alone (Fig. 5f). Given the benefits of combining TRAIL with natural products to enhance tumour cell apoptosis *in vitro* in our current study (Fig. 2e), along with results by others^22^, we assessed whether administration of the natural product resveratrol could combine with our approach to further increase TRAIL-mediated tumour apoptosis *in vivo*. Resveratrol is a naturally occurring polyphenol that exhibits numerous health benefits including anti-inflammatory, antioxidant and antitumour activities, and has previously been shown to increase TRAIL-mediated apoptosis in solid tumours^49^. In combination with resveratrol, amplification of TRAIL apoptosis using our particle-based approach reduced tumour growth by over 80% compared with control mice (Fig. 5f). These combined results suggest that the targeted polymeric particles can be utilized to mechanically amplify TRAIL-mediated apoptosis in solid tumour models, and can be combined with natural products to further reduce tumour growth.

**Figure 6:**
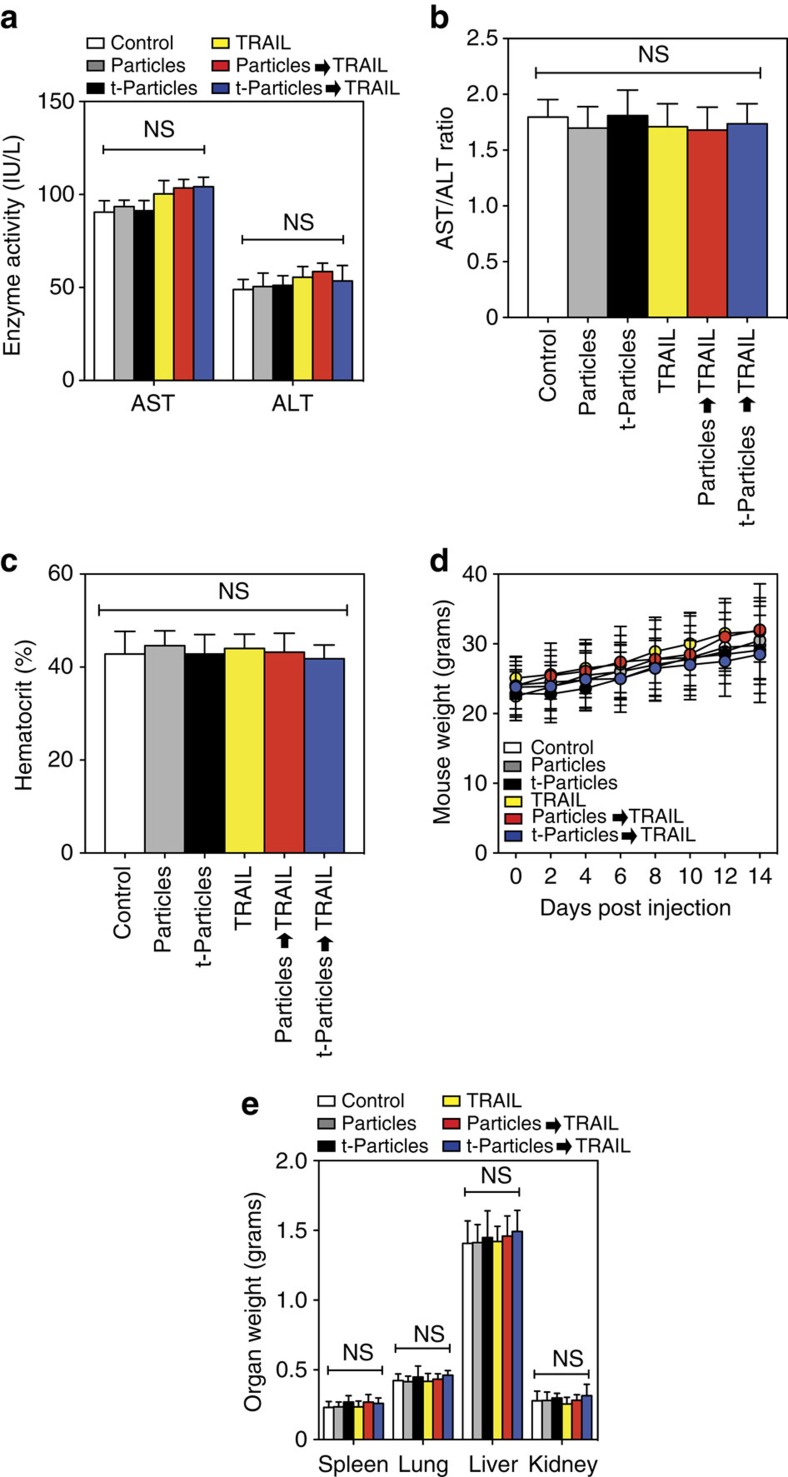
Delivery of nontargeted particles (Particles) and EpCAM-targeted PLGA particles (t-Particles) followed by TRAIL therapeutic does not affect non-target cells and tissues *in vivo*. (**a**) Serum levels of aspartate aminotransferase (AST) and alanine aminotransferase (ALT) liver enzymes in mice from controls and different treatment groups at the end of the 2-week study. (**b**) AST/ALT ratio in serum of mice from controls and different treatment groups at the end of the 2-week study. AST/ALT >2 indicates liver toxicity. (**c**) Haematocrit levels of mice from controls and different treatment groups. Blood was drawn before killing of mice. (**d**) Weight of mice 0–2 weeks post injection of TRAIL and particle-functionalized tumour cells. (**e**) Weight of excised organs post-mortem. Data are reported as mean±s.e. Different treatment groups were compared for statistical significance using a one-way analysis of variance (ANOVA) for multiple comparisons. NS, not significant. *N*=5 mice for all treatments.

Toxicity results showed that treated mice exhibited no evidence of elevated liver enzymes in serum (Fig. 6a,b), no significant differences in haematocrit relative to untreated mice (Fig. 6c), no loss of appetite or body weight or behavioural distress compared with untreated mice (Fig. 6d) and no enlarged kidney mass (Fig. 6e). It is important to note that the TRAIL dosage used to target tumour cells in the bloodstream is approximately two orders of magnitude less than the concentrations shown to be well tolerated in previous animal and human trials with soluble TRAIL^22^. Collectively, these results suggest that targeted polymeric particles delivered and bound to tumour cells *in vivo* can leverage physical forces to increase the apoptotic effect of TRAIL, with negligible off-target toxicity.

## Discussion

In summary, we have demonstrated that functionalization of the tumour cell surface with polymeric particles can amplify TRAIL-mediated tumour cell killing in the presence of fluid shear stress both *in vitro* and *in vivo* in mice. Building upon our initial finding that tumour cells, but not normal cells, show increased sensitivity to TRAIL-mediated apoptosis under fluid shear stress conditions of the circulation, we found that both degradable and nondegradable particles functionalized to the tumour cell surface act as mechanical amplifiers of this response. This approach reduced the viability of TRAIL-resistant tumour cells when treated with the ligand, and further increased tumour cell killing in combination with the natural product piperlongumine. Similar functionalization did not have toxic effects on normal human leukocytes and human endothelial cell monolayers. Tumour cells exhibited increased TRAIL-mediated apoptosis proportional to higher shear stress in a manner that was not recapitulated with doxorubicin treatment, indicating that this response is receptor-mediated apoptosis specific. Furthermore, functionalization of tumour cells with increased number and size of polymeric particles enhanced TRAIL-mediated apoptosis under shear conditions, but not static conditions, suggesting that increased force exerted on the cell membrane enhanced the apoptotic effect. Mechanical amplification of tumour cell killing was dependent on caspase signalling and enhanced TRAIL death receptor expression on the tumour cell surface, suggesting that forces exerted by polymeric particles increased the therapeutic effect of TRAIL.

Under varying physical forces exhibited *in vivo*, treatment of tumour cells with an EpCAM-targeted PLGA particle platform enhanced a subsequent injection of soluble TRAIL delivered via the circulation, and reduced both circulating tumour cells in blood and overall tumour cell burden by over 90%. In a PC-3 xenograft model, a significant decrease in tumour growth was observed in mice treated with particles followed by TRAIL, compared with TRAIL treatment alone. In combination with the natural product resveratrol, administration of targeted particles followed by TRAIL reduced tumour growth by 80% compared with controls. It is important to note that the half-life of TRAIL is a major obstacle to the clinical application of the ligand, which was measured to be <30 min in animal studies^50^. However, recent advances via conjugation with PEG, human serum albumin and lipid nanoparticles have vastly improved the pharmacokinetic profile of TRAIL^51^,^52^,^53^,^54^. Given the promising results shown in this study with unmodified soluble TRAIL, we envision that our approach can be combined with engineered TRAIL nanoparticles and conjugates to show increased efficacy. These results highlight a potential new role for polymeric nanomaterials, traditionally used as drug delivery vehicles, as amplifiers of receptor-mediated signalling and function in the presence of physical forces. Based on this collective evidence, a broad range of micro- and nanotechnology platforms can potentially be applied to enhance receptor–ligand interactions and signalling.

## Methods

### Reagents and antibodies

RPMI-1640 cell culture medium, McCoy's 5A modified medium, Dulbecco's modified Eagle's medium/F-12 cell culture medium, fetal bovine serum (FBS), penicillin–streptomycin (PenStrep), phosphate-buffered saline (PBS), trypsin-EDTA solution, NHS–PEG_12_–biotin and Pierce D-Luciferin, monosodium salt were all purchased from Invitrogen (Grand Island, NY, USA). Maleimide-(PEG)-amine (MW 1000) was purchased from Nanocs Inc. (New York, NY, USA). Eagle's minimum essential medium, recombinant human insulin and Trypan blue solution were purchased from Thermo Scientific (Boston, MA, USA). Sodium hydroxide (NaOH), bovine serum albumin (BSA), biotin, 2-(*N*-morpholino)ethanesulfonic acid (MES) solution and 1-(3-dimethylaminopropyl)-3-ethylcarbodiimide (EDAC) were obtained from Sigma-Aldrich (St Louis, MO, USA). Recombinant human TRAIL was obtained from R&D Systems (Minneapolis, MN, USA). Doxorubicin hydrochloride was purchased from Sigma-Aldrich. Resveratrol was purchased from LKT Laboratories, Inc. (St Paul, MN, USA). Piperlongumine was purchased from Cayman Chemical Company (Ann Arbor, MI, USA). CellTiter-Glo Luminescent Cell Viability Assay was purchased from Promega (Promega Corporation, Madison, WI, USA). A Dead Cell Apoptosis Kit consisting of an annexin-V fluorescein isothiocyanate (FITC) or allophycocyanin (APC) conjugate and PI was purchased from Invitrogen. Fluorescent and nonfluorescent streptavidin-functionalized PS particles (diameters: 100–1,000 nm) were purchased from Bangs Laboratories, Inc. (Fishers, IN, USA). Degradable PLGA particles (diameter: 500–1000, nm) surface functionalized with carboxylic acid (COOH) were purchased from Phosphorex Inc. (Hopkinton, MA, USA). Mouse monoclonal anti-human EpCAM (clone 158210) and EpCAM-APC (clone 158206) antibodies were purchased from R&D Systems (Minneapolis, MN, USA). Mouse anti-human TRAIL DR4 (clone DJR1) and DR5 (clone DJR2-2) antibodies were purchased from Biolegend (San Diego, CA, USA).

### Cell culture and cell lines

Colon cancer cell line COLO 205 (ATCC number CCL-222) was obtained from ATCC (Manassas, VA, USA) and cultured in RPMI-1640 medium supplemented with 10% (vol/vol) FBS and 1% (vol/vol) PenStrep. A dual-labelled luciferase and GFP+ COLO 205 colon cancer cell line was obtained from Genecopoeia (Rockville, MD, USA) and cultured in RPMI-1640 supplemented with 10% (vol/vol) FBS and 1% (vol/vol) PenStrep. Prostate cancer cell line PC-3 (ATCC number CRL-1435) was obtained from ATCC and cultured in Dulbecco's modified Eagle's medium supplemented with 10% (vol/vol) FBS and 1% (vol/vol) PenStrep. Breast cancer cell line MCF7 (ATCC number HTB-22) was obtained from ATCC and cultured in Eagle's minimum essential medium supplemented with 10% (vol/vol) FBS, 1% (vol/vol) Penstrep and 0.01 mg ml^−1^ human recombinant insulin. Colon cancer cell line HT29 (ATCC number HTB-38) was obtained from ATCC and cultured in McCoy's 5A modified medium supplemented with 10% (vol/vol) FBS and 1% (vol/vol) Penstrep. Human umbilical vein endothelial cells (HUVECs) were purchased from Cascade Biologics and maintained in Medium 200 (Cascade Biologics) supplemented with low-serum growth supplement (Cascade Biologics) and 5% FBS (Invitrogen). HUVECs from passages 2–5 were used for experiments. All cell lines were cultured under humidified conditions at 37 °C and 5% CO_2_. For all experiments, >95% viability was assessed using a Trypan blue exclusion dye.

### Mononuclear leukocyte isolation

Mononuclear leukocytes were isolated from human peripheral blood collected from healthy donors that was purchased from Research Blood Components (Boston, MA, USA), and was approved by the MIT Institutional Animal Care and Use Committee^19^. Human blood was layered over 1-Step Polymorphs human cell separation media (Accurate Chemical & Scientific Corporation, Westbury, NY, USA), and leukocytes were isolated from blood via centrifugation at 480 × *g* for 50 min at 23 °C in a Marathon 8K centrifuge (Fisher Scientific, Pittsburgh, PA, USA). Leukocytes were extracted and washed in Ca^2+^ and Mg^2+^-free Hank's balanced salt solution (HBSS), and all remaining red blood cells in the suspension were lysed hypotonically. Leukocytes were resuspended at a concentration of 0.5 × 10^6^ cells per ml in HBSS containing 0.5% human serum albumin, 2 mM Ca^2+^, 1 mM Mg^2+^ and 10 mM HEPES (Invitrogen), buffered to pH 7.4, before functionalization with particles. For all leukocyte experiments, >95% viability was assessed using a Trypan blue exclusion dye.

### Endothelial cell and HT29 tumour cell monolayer growth

Endothelial cell (EC) and HT29 tumour cell monolayers were grown on circular glass coverslips before functionalization with particles^55^,^56^. Then, 40 mm diameter circular glass coverslips (Thermo Scientific) were plasma treated (Harrick Plasma Cleaner) for 2 min and subsequently incubated in 1% polyethylenemine at room temperature (RT) for 10 min. Coverslips were then washed in deionized water three times and treated with 0.1% glutaraldehyde (Sigma-Aldrich) in PBS at RT for 30 min. Coverslips were washed in deionized water three times, dried and treated with 0.1 mg ml^−1^ type I rat-tail collagen (Becton Dickinson) in HEPES (pH 8.0; Sigma-Aldrich) for 2 h at 4 °C. Coverslips were placed in Petri dishes (60 mm × 15 mm; Sigma-Aldrich), washed three times in PBS and briefly sterilized via ultraviolet exposure for 15 min. HUVECs were plated on coverslips, at a density of 500,000 cells per coverslip, in Medium 200 supplemented with low-serum growth supplement (Cascade Biologics), 5% FBS (Invitrogen) and 100 U ml^−1^ PenStrep. HT29 cells were plated at a density of 500,00 cells per coverslip in McCoy's 5A modified medium supplemented with 10% (vol/vol) FBS and 1% (vol/vol) Penstrep. Both cell types were cultured for ∼4 days on coverslips before functionalization with particles.

### Polymeric particle conjugation to normal and tumour cells

Mononuclear leukocytes, PC-3 and COLO 205 tumour cells were washed in Mg^2+^ and Ca^2+^-free HBSS (Invitrogen, Carlsbad, CA, USA) followed by treatment with trypsin-EDTA for 5–10 min at 37 °C before handling. EC and HT29 monolayers, in addition to tumour cells and leukocytes, were treated with NHS–PEG–biotin at a concentration of 1 μM for 30 min at RT, washed and treated with streptavidin-functionalized PS particles at a ratio of 1:480 particles per cell. Samples were subjected to rocking at 4 °C for 1 h, and washed before use in assays. For biodegradable particle functionalization used for *in vitro* and *in vivo* assays, 10 mg of PLGA-COOH particles (Phosphorex; diameter: 500 nm) were resuspended in 0.2 ml 50 mM MES buffer (pH 5.2), vortexed and treated with 0.02 ml EDAC solution (concentration: 200 mg ml^−1^). Then, 0.5 mg of maleimide-PEG-NH_2_ was added to the particle solution and gently mixed at RT for 4 h. Particle solution was washed three times, followed by overnight incubation and gentle mixing with 0.5 mg of a thiolated anti-EpCAM)antibody. Anti-EpCAM was thiolated using 1 mM Traut's reagent following a protocol supplied by the manufacturer. Excess Traut's reagent was removed using a desalting column. Particles were subsequently washed via centrifugation and resuspended in PBS containing 0.5% BSA. For *in vitro* assays, cells treated with PLGA particles were subjected to rocking at 4 °C for 1 h, and washed before use in assays.

To determine particle internalization, tumour cells were functionalized with 0.5 μM fluorescent Fluoresbrite YG (yellow–green; *λ*_ex_, 445 nm; *λ*_em_, 500 nm) PS particles and subsequently washed with a 0.4 % (w/v) Trypan blue solution^57^ to quench extracellular fluorescence. The percentage of cells with internalized particles was assessed using confocal microscopy. To determine the role of PEG linkers in mediating stable conjugation of particles to the tumour cell surface, cells were treated with and without PEG linkers and subsequently treated with fluorescent PS particles (ratio: 120 particles per cell). Cells were then washed three times in PBS at 300 × *g* for 5 min and subsequently treated with an anti-EpCAM antibody for 45 min at 4 °C. Cells were then washed in PBS and fluorescent particle functionalization to EpCAM+ cells was determined using a BD fluorescence-activated cell sorter (FACS) Canto (BD Biosciences, San Jose, CA, USA).

To quantify unbound particles post functionalization, cell samples were centrifuged and the supernatant containing unbound fluorescent particles was collected. Supernatant fluorescence was quantified using a fluorescent plate reader, and was then compared with cell supernatant in the absence of particles, as well as a fluorescent particle sample as a positive control (Supplementary Fig. 4). The fluorescence distribution of cells functionalized with particles was measured using flow cytometry.

### *In vitro* fluid shear stress assays

Functionalized and nonfunctionalized tumour cells, leukocytes and EC or HT29 monolayers were exposed to fluid shear stress using a cone-and-plate viscometer (Brookfield, Middleboro, MA, USA) consisting of a stationary plate underneath a rotating cone maintained at 37 °C by a circulating water bath^25^. The cone-and-plate device design allows for a uniform shear rate to be applied to cells^58^. The shear rate (*G*) does not depend on distance from the centre of the cone, and is given by:


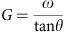


where *ω* is the cone angular velocity (rad s^−1^) and *θ* is the cone angle (rad). A laminar flow field is expected under all experimental conditions. For a Newtonian fluid under these conditions, the shear stress, *τ* , is proportional to the shear rate being applied:


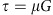


where μ is the viscosity of the medium.

Before fluid shear stress exposure, the stationary plate and rotating cone were washed thoroughly with 70% ethanol, followed by incubation with 5% BSA in PBS to prevent nonspecific cell adhesion to the cone and plate surfaces. Tumour cells and human leukocytes at a density of 0.5 × 10^6^ cells per ml in serum-free medium were treated with TRAIL and immediately exposed to fluid shear stress (0.05–12.0 dyn cm^−2^) in the viscometer. The shear stress range was chosen based on its physiological relevance. High shear stress values (∼30 dyn cm^−2^) initially investigated were not pursued further because of insufficient cell recovery (Supplementary Fig. 11). Shear stress exposure times for COLO 205 and PC-3 tumour cells were 30 and 60 min, respectively. Effective TRAIL dosages and incubation times to initiate the apoptotic process were verified using a caspase-8 activity assay, which showed increased caspase-8 activity in COLO 205 tumour cells after incubation with TRAIL under static conditions for 30 and 60 min (Supplementary Fig. 2). Coverslips coated with HUVEC or HT29 monolayers were adhered to the plate of a cone-and-plate viscometer using vacuum grease and were then exposure to fluid shear stress for 90 min and 6 h, respectively. For combination therapies, TRAIL-resistant HT29 cells were treated simultaneously with 0.1 μg ml^−1^ TRAIL and 15 μM piperlongumine in the presence of shear stress. After exposure to shear stress, cells were washed in PBS, resuspended in culture medium, incubated overnight (∼10 h) and assessed for cell viability using a CellTiter-Glo luminescent viability assay. To assess the mode of cell death, cells were washed in PBS after shear stress exposure, resuspended in culture medium, incubated for 4 h and subsequently analysed for mode of cell death using an annexin-V Apoptosis kit. For receptor expression assays, cells were labelled with anti-human TRAIL DR4 and DR5 antibodies immediately after both TRAIL and shear stress exposure, and assessed for DR4 and DR5 expression using flow cytometry. For caspase inhibition studies, cells were treated with 50 μM of pan-caspase inhibitor Z-VAD-FMK, caspase-8 inhibitor Z-IETD-FMK or caspase negative control inhibitor Z-FA-FMK (R&D Systems) for 4 h at 37 °C before TRAIL treatment. For doxorubicin studies, cells were treated with doxorubicin (concentration: 20 μM) for 2 h in the presence of shear stress. The doxorubicin dosing regimen and incubation time was previously shown to induce apoptosis in tumour cells^59^. After treatment, cells were washed thoroughly in PBS and resuspended in culture medium for overnight incubation (12 h) before cell viability analysis.

### Brightfield and phase-contrast microscopy

Cell samples were washed and placed into 24-well plates after treatment. Samples were incubated at 37 °C for 60 min to allow cells to adhere to the plate. Images were captured via brightfield and phase-contrast microscopy using an EVOS microscope (Invitrogen, Grand Island, NY, USA) to observe the presence of viable cells and membrane blebbing that is characteristic of cellular apoptosis. Images were processed using ImageJ software (US National Institutes of Health, Bethesda, MD, USA).

### Cell viability and apoptosis assays

A CellTiter-Glo solution was prepared using the manufacturer's protocol and was used to detach cells from the surface of the plate. Equal volumes of CellTiter-Glo solution was added to each sample volume, and sample aliquot triplicates were plated on black, flat bottom, chimney well 96-well plates (Greiner Bio-One, Monroe, NC, USA). Luminescence based on ATP concentration was measured using a Tecan Infinite M200 Pro plate reader (Research Triangle Park, NC, USA). FITC- and APC-conjugated annexin-V assays (Trevigen, Gaithersburg, MD, USA) were used to assess cell apoptosis and necrosis. The manufacturer's instructions were followed to prepare samples for flow cytometric analysis. Viable cells were identified as being negative for both annexin-V and PI, early apoptotic cells were positive for annexin-V only, late apoptotic cells were positive for both annexin-V and PI and necrotic cells were positive for PI only.

### Caspase-8 activation assays

A caspase-8 fluorometric assay was prepared using the manufacturer's protocol (R&D Systems) and was used to detect caspase-8 activation in cell samples. Equal numbers of cells from all samples were lysed and place into flat-bottom 96-well plates. Cell lysate was incubated with reaction buffer and caspase-8 fluorogenic substrate in a 1:1 volume ratio, and incubated at 37 °C for 1 h. Caspase-8 activity was measured using a Tecan Infinite M200 Pro plate reader using filters that allow light excitation at a wavelength of 400 nm and collect emitted light at a wavelength of 505 nm.

### *In vivo* COLO 205 tumour cell studies

Female nu/nu mice aged 16–20 weeks, weighing 25–32 *g*, were obtained from The Jackson Laboratory (Bar Harbor, ME, USA). Mice were restrained, and luciferase+, GFP+ COLO 205 tumour cells were injected via tail vein (2 × 10^6^ cells in 50 μl) using a 30-guage needle (Becton Dickson, Rutherford, NJ, USA). Mice were then injected with nontargeted and EpCAM-targeted PLGA particles (500 nm diameter; ∼500 particles per tumour cell) 15 min post tumour cell injection. At 30 min post particle injection, mice were injected with 100 μl of saline or soluble TRAIL (sTRAIL; 1.5 μg ml^−1^; TRAIL plasma concentration ∼0.1 μg ml^−1^) via tail vein using a 30-gauge needle. Five mice were used in each group. All animal procedures were approved by the MIT institutional animal care and use committee.

### Analysis of tumour cells in bloodstream

Blood (∼200 μl) was collected into sodium heparin-coated tubes (Becton Dickinson) via submandibular bleed using a sterile lancet (Medipoint, Mineola, NY, USA) 90 min post injection of TRAIL. Circulating COLO 205 cells were separated from whole blood using Ficoll-Paque PLUS (Amersham Biosciences, Piscataway, NJ, USA) as per the manufacturer's instructions. Briefly, blood samples were carefully layered over 1.5 ml of Ficoll and centrifuged at 480 × *g* for 50 min at RT. After centrifugation, the buffy coat was collected, washed in buffer containing Ca^2+^ and cultured for 4–6 h in multiwell plates in culture medium. GFP+ COLO 205 cells isolated from whole blood were assessed using flow cytometry. To determine mode of cell death of tumour cells in the blood stream, tumour cells were separated from whole blood, cultured for ∼2 h in culture medium and labelled with an APC-conjugated annexin-V apoptosis kit for flow cytometry analysis.

### Whole-body BLI of tumour cell burden in mice

Whole-body BLI was performed using an IVIS Spectrum-bioluminescent and fluorescent imaging system (Xenogen Corporation, Waltham, MA, USA) consisting of CCD camera mounted in a light-tight specimen box. Images were captured and BLI signals were quantified using Living Image acquisition and analysis software (Xenogen). Anaesthetized mice were placed in the IVIS Imaging System and images were captured from ventral views 10–15 min after intraperitoneal injection of D-luciferin (150 mg kg^−1^ body weight). Successful tail vein injections of tumour cells were determined using images showing systemic bioluminescence signal distributed throughout mice. Mice with evidence of a satisfactory injection were used for further imaging time points. Tumour cell signals within mice were monitored *in vivo* at 1 and 2 weeks post injection.

### PC-3 xenograft studies

Nu/nu mice (6 weeks, Charles River Laboratories) were subcutaneously inoculated in the back with 3 × 10^6^ PC-3 tumour cells. Mouse tumour size was measured using a vernier caliper and tumour volume (*V*) was calculated as *V*=*L* × *W*^2^/2, where *L* and *W* were the length and width of the tumour, respectively. Tumour-bearing mice were weighed and randomly divided into six different groups of 5 mice per group when the tumour volume reached 100 mm^3^. The following treatment protocol was implemented over a 21-day period: *Group 1* (*Control*), vehicle control (0.1 ml PBS) administered through oral gavage 3 times per week starting at day 0, along with another vehicle control (0.1 ml PBS) administered intravenously every 4 days, starting at day 1; *Group 2* (*Resveratrol*), resveratrol (30 mg kg^−1^, in 0.1 ml PBS) administered through gavage 3 times per week; *Group 3* (*TRAIL*), soluble TRAIL (15 mg kg^−1^ in 0.1 ml PBS) administered intravenously every 4 days, starting at day 1; *Group 4* (*t-Particles*→*TRAIL*), EpCAM-targeted PLGA particles (size: 500 nm) administered intravenously followed by soluble TRAIL (15 mg kg^−1^ in 0.1 ml PBS) 3 h later, administered intravenously every 4 days starting at day 1; *Group 5* (*TRAIL+Resveratrol*), resveratrol administered through gavage, and TRAIL administered intravenously; and *Group 6* (*t-Particles*→*TRAIL+Resveratrol*), resveratrol administered through gavage, targeted particles administered intravenously followed by TRAIL 3 h later. Mice were housed under pathogen-free conditions.

### *In vivo* particle imaging study

EpCAM-targeted FITC-fluorescent PLGA particles (size: 500 nm) were administered intravenously into nu/nu mice (dose: 40 mg kg^−1^) when tumour volumes reached 200 mm^3^. PLGA particles are biodegradable and do not induce toxicity, as dosages as high as 500 mg kg^−1^ have previously been shown to not alter animal weight^60^. At 3 h post injection, particle biodistribution was detected via *in vivo* fluorescence imaging using an IVIS imaging system. After *in vivo* imaging, mice were killed. Tumours, spleen, kidneys, lungs, heart and liver were then harvested and assessed for particle fluorescence via *ex vivo* fluorescence imaging.

### *In vivo* toxicity

Given that hepatocytes have shown sensitivity to TRAIL at high dosages, serum levels of alanine aminotransferase (ALT) and aspartate aminotransferase (AST), which indicate leakage from damaged hepatocytes because of inflammation or apoptosis, were quantified to assess liver function in treated mice. ALT and AST are normally stored within hepatocytes, and damage to hepatocytes can induce elevated ALT and AST levels in serum. Serum levels of ALT and AST were analysed using a colorimetric assay kit (BioVision). To quantify potential changes in haematocrit, blood was drawn via cardiac puncture using a 28G heparin-coated syringe and collected in a heparinized tube. Two drops per animal were placed in haematocrit tubes and spun for 2 min using a CritSpin Microhematocrit Centrifuge. The ratio of red blood cells to total blood volume was used to calculate haematocrit levels. All mice were weighed at least once per week throughout the duration of the experiment. All organs were weighed upon conclusion of the experiment.

### Statistical analysis

All data sets were analysed using GraphPad Prism 5.0 (GraphPad software, San Diego, CA, USA) and Microsoft Excel (Microsoft, Redmond, WA, USA).

### Data availability

All data and/or analysis tools that support the conclusions of this study are available on request from the authors.

## Additional information

**How to cite this article:** Mitchell, M. J. *et al*. Polymeric mechanical amplifiers of immune cytokine-mediated apoptosis. *Nat. Commun.* **8,** 14179 doi: 10.1038/ncomms14179 (2017).

**Publisher's note:** Springer Nature remains neutral with regard to jurisdictional claims in published maps and institutional affiliations.

## Supplementary Material

Supplementary Information Supplementary figures.
