# Supplementary material for: Polymeric mechanical amplifiers of immune cytokine-mediated apoptosis
Source: Nat Commun. 2017 Mar 20;8:14179. doi: 10.1038/ncomms14179 (PMC5364380; doi:10.1038/ncomms14179)
Supplement: Supplementary Information — Supplementary figures. [file ncomms14179-s1.pdf]

Supplementary Figures:

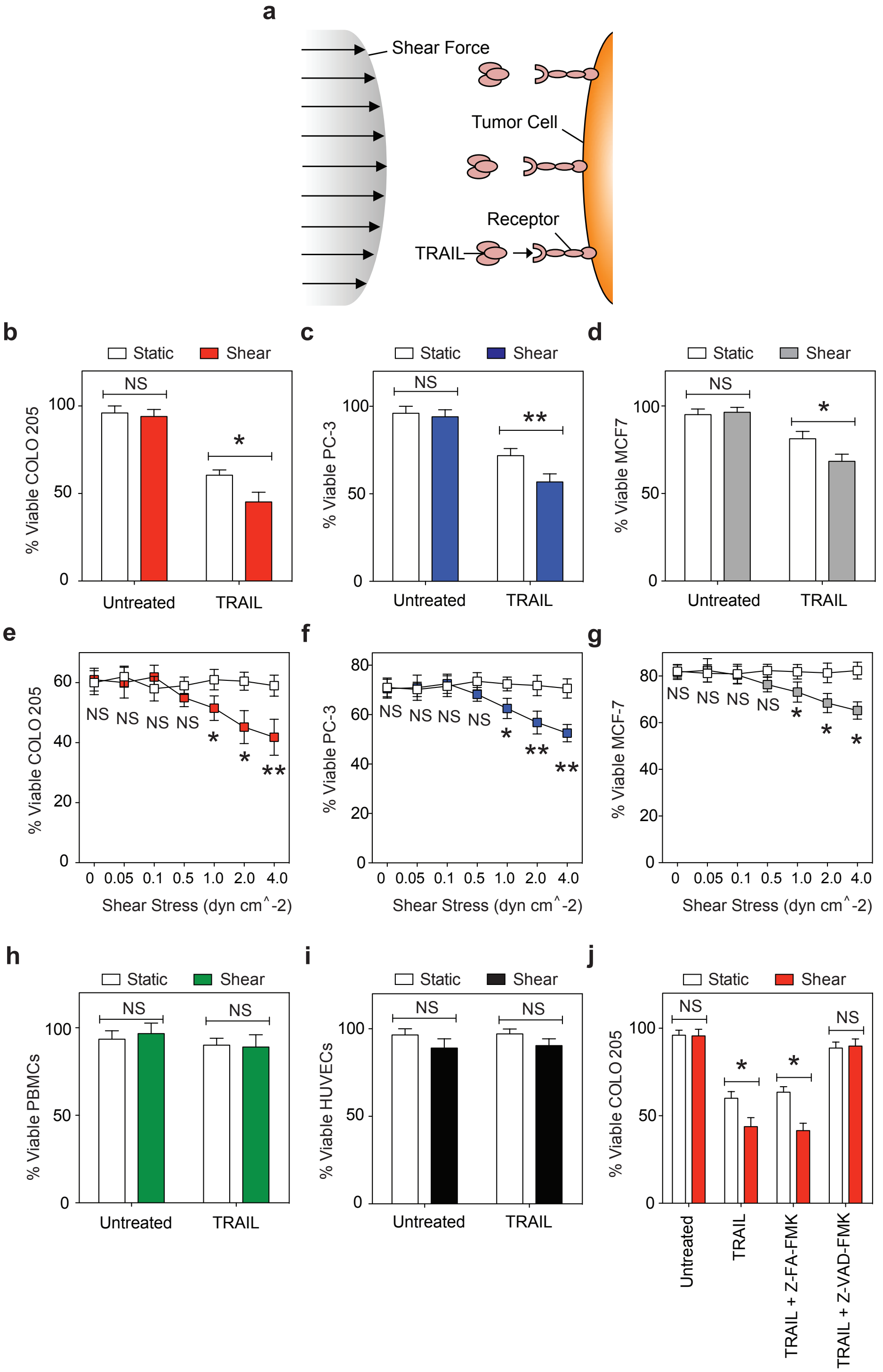

Supplementary Figure 1: TRAIL-mediated apoptosis in colon, breast, and prostate tumor cells is enhanced under fluid shear stress exposure, while normal cells remain unaffected. (**a**) Schematic of TRAIL-mediated tumor cell apoptosis in presence of a fluid shear force. (**b-d**) Colon cancer COLO 205 (**b**), prostate cancer PC-3 (**c**), and breast cancer MCF7 (**d**) cell viability after exposure to TRAIL (0.1 μg mL^-1) under shear (2.0 dyn cm^-2) and static conditions. COLO 205, PC-3, and MCF7 were treated

with TRAIL under shear and static conditions for 30, 60, and 90 min, respectively. Samples were then washed and incubated overnight, and assessed for cell viability using a CellTiter-Glo® luminescence assay. (**e-g**) COLO 205 (**e**), PC-3 (**f**), and MCF7 (**g**) tumor cell viability after exposure to TRAIL (0.1 μg mL^-1) under a range of shear stress conditions (0.05-4.0 dyn cm^-2). COLO 205, PC-3, and MCF7 were treated with TRAIL under shear and static conditions for 30, 60, and 90 min, respectively. (**h,i**) Human peripheral blood mononuclear cell (PBMC) (**h**), and human umbilical vein endothelial cell (HUVEC) (**i**) viability after exposure to TRAIL (0.1 μg mL^-1) under shear (2.0 dyn cm^-2) and static conditions. PBMCs and HUVEC monolayers grown on coverslips were treated with TRAIL under shear and static conditions for 90 min. Samples were then washed and incubated overnight, and assessed for cell viability using a CellTiter-Glo® luminescence assay. (**j**) Fluid shear stress sensitization to TRAIL-mediated apoptosis is caspase-dependent. COLO 205 tumor cells were treated with 0.1 μg mL^-1 TRAIL, negative control inhibitor Z-FA-FMK followed by 0.1 μg mL^-1 TRAIL, and pan caspase inhibitor Z-VAD-FMK followed by 0.1 μg mL^-1 TRAIL, exposed to static and shear (2.0 dyn cm^-2) for 30 min. Samples were then washed and incubated overnight, and assessed for cell viability using a CellTiter-Glo® luminescence assay. Data are reported as the mean ± s.e. Different treatment groups were compared for statistical significance using a Student's two-tailed t-test. ***P*<0.01. **P*<0.05. NS: not significant. These data suggest that fluid shear stress exposure increases TRAIL-mediated apoptosis in breast, prostate, and colon tumor cells, but not normal cells. Experiments were repeated 5 times.

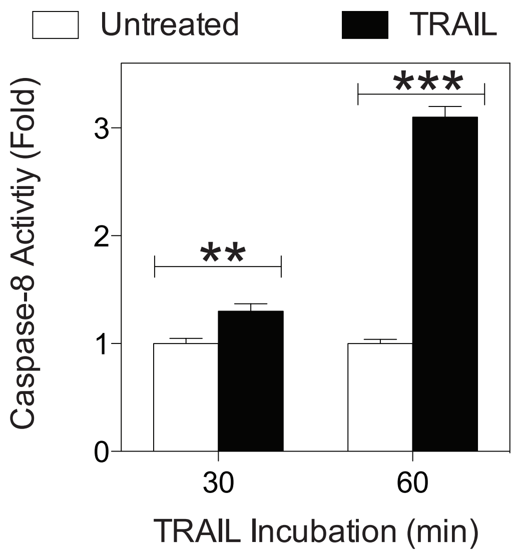

__Supplementary Figure 2:__ Caspase-8 activation in COLO 205 tumor cells after incubation with TRAIL. COLO 205 cells were incubated with TRAIL (0.1 μg mL^-1) for 30 and 60 min and were then assessed for caspase-8 activity, an early indicator of apoptosis, using a caspase-8 fluorometric assay kit (R&D Systems). All treatments were normalized to untreated controls. These findings indicate that caspase-8 activity in TRAIL-treated COLO 205 tumor cells can be measured as early as 30 min post-treatment. Data are reported as the mean ± s.e. Different treatment groups were compared for statistical significance using a Student's two-tailed t-test. ***P*<0.01. **P*<0.05. Experiments were repeated 3 times.

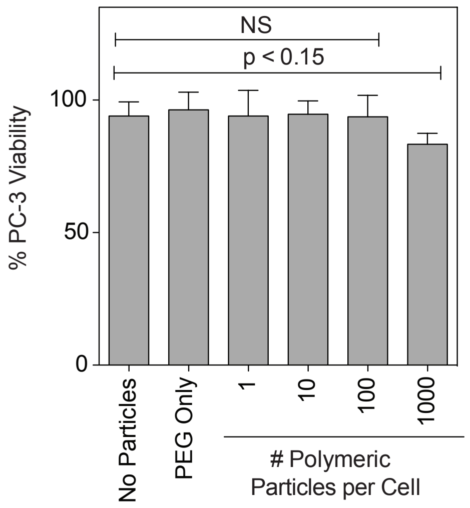

<u>Supplementary Figure 3:</u> Functionalization of tumor cell surface with polymeric particles does not affect tumor cell viability. Prostate PC-3 tumor cells were functionalized with PEG-NHS and increasing numbers of polymeric polystyrene particles (500 nm diameter) per tumor cell. Samples were incubated overnight, and assessed for cell viability using a CellTiter-Glo® luminescence assay. NS: not significant. These data suggest that polymeric particles conjugated to the tumor cell surface using PEG-NHS linkers do not significantly affect tumor cell viability. Data are reported as the mean ± s.e. Different treatment groups were compared for statistical significance using a one-way analysis of variance (ANOVA). NS: not significant. Experiments were repeated 5 times.

a

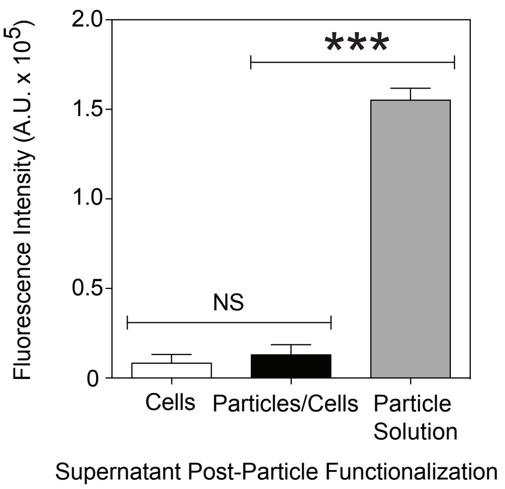

b

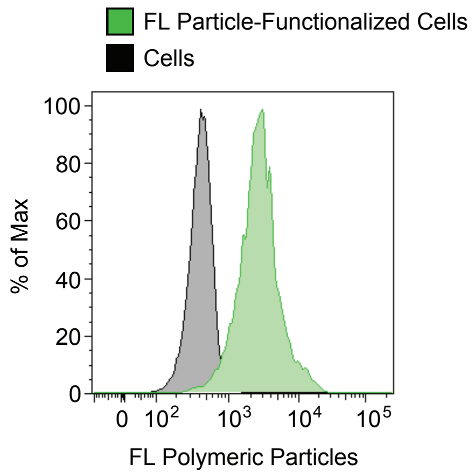

Supplementary Figure 4: Polymeric particles tether to the tumor cell surface with minimal particles remaining in solution. (**a**) Fluorescence intensity measurements of cell supernatants after treatment with fluorescent particles. PC-3 tumor cells in suspension were treated with NHS-PEG-biotin at a concentration of 1 μM for 30 min at RT, washed, and then treated with streptavidin-functionalized, fluorescent (FL) polystyrene particles at a ratio of 240 particles per cell. Samples were subjected to rocking at 4°C for 1 h, and centrifuged to remove any unbound fluorescent particles. Supernatant was collected and fluorescence was measured using a plate reader. Particle solution denotes the fluorescence of a particle sample before functionalization to cells. (**b**) Flow cytometry fluorescence measurements of tumor cells functionalized with fluorescent particles. Normal Gaussian distribution indicates that particles do not preferentially bind to subsets of PC-3 tumor cells. These data suggest that particles bind to the tumor cell surface within 1 h of incubation with negligible particles remaining, and that the majority of the tumor cell population is uniformly functionalized with particles. Data are reported as the mean ± s.e. Different treatment groups were compared for statistical significance using a Student's two-tailed t-test. ***P<0.001. NS: not significant. Experiments were repeated 4 times.

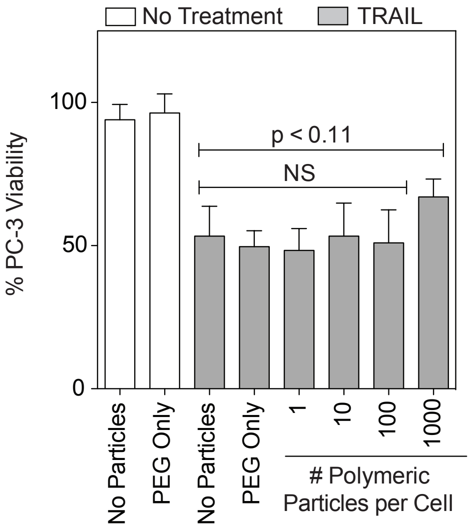

Supplementary Figure 5: Polymeric particles conjugated to the tumor cell surface do not interfere with TRAIL-mediated apoptosis. Prostate PC-3 tumor cells were functionalized with NHS-PEG-biotin and increasing numbers of polymeric polystyrene particles (1-1000 particles per cell; 500 nm particle diameter) per tumor cell. Tumor cells were subsequently treated with 0.1 μg/mL TRAIL for 1 h, washed, incubated overnight, and assessed for cell viability using a CellTiter-Glo® luminescence assay. Data are reported as the mean ± s.e. Different treatment groups were compared for statistical significance using a one-way analysis of variance (ANOVA) for multiple comparisons. NS: not significant. This finding suggests that polymeric particles can be conjugated to the tumor cell surface without significantly altering TRAIL-mediated apoptosis. Experiments were repeated 5 times.

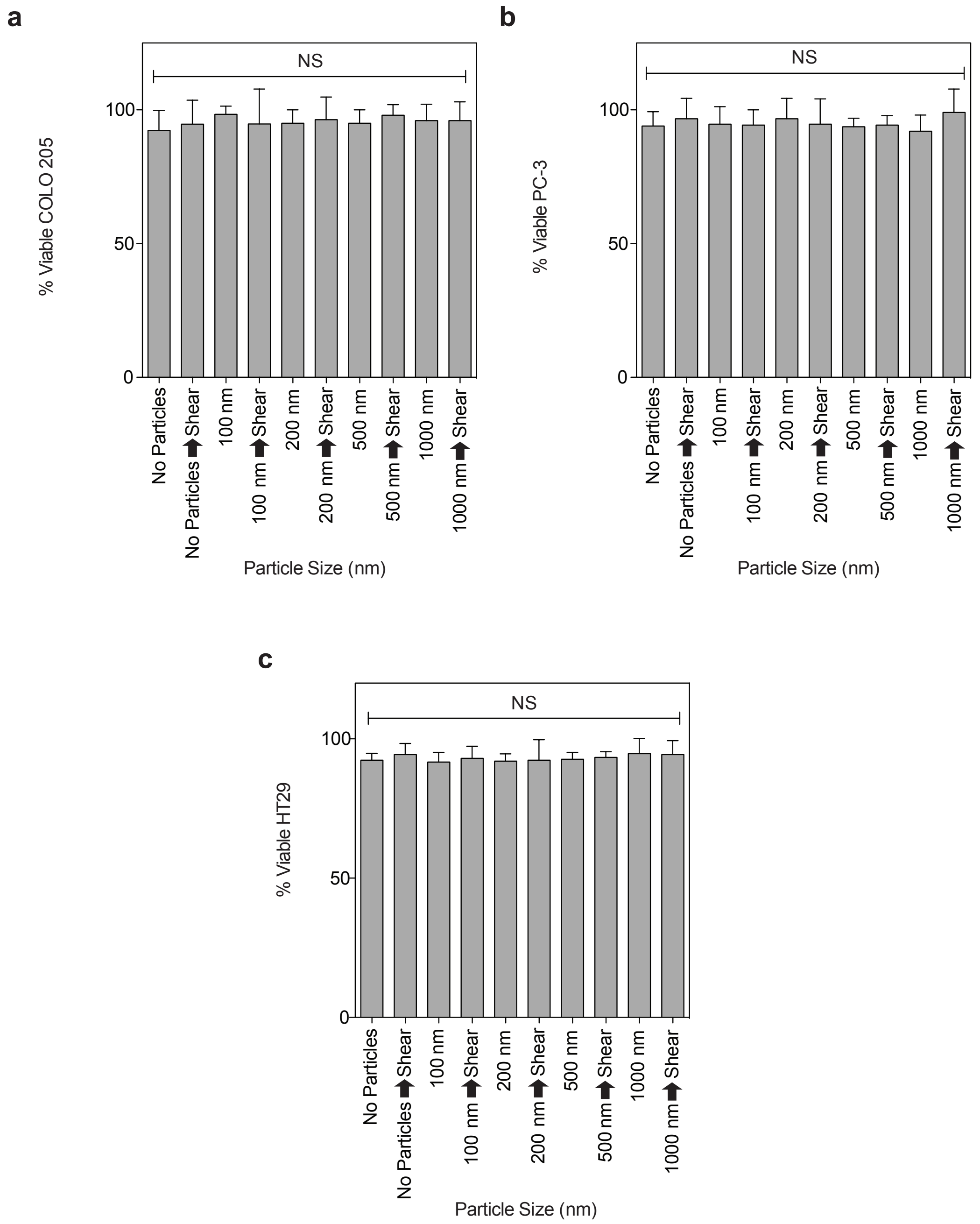

Supplementary Figure 6: Particle functionalization of the tumor cell surface does not affect cell viability after exposure to fluid shear stress. (**a-c**) Colon COLO 205 (**a**), prostate PC-3 (**b**), and TRAIL-resistant colon HT29 (**c**) tumor cells were functionalized with polymeric polystyrene particles of varying sizes (100-1000 nm diameter) and exposed to a fluid shear force (shear stress: 4.0 dyn cm^-2). COLO 205, PC-3, and HT29 tumor cells were exposed to shear for 30 min, 1 h, and 6 h respectively. Tumor cells were then washed, incubated overnight, and assessed for cell viability using a CellTiter-Glo® luminescence assay. Data are reported as the mean ± s.e. Different treatment groups were compared for statistical significance using a one-way analysis of variance (ANOVA). NS: not significant. These data suggest that polymeric particles conjugated to the tumor cell surface do not significantly affect cell viability in the presence of fluid shear stress. Experiments were repeated 5 times.

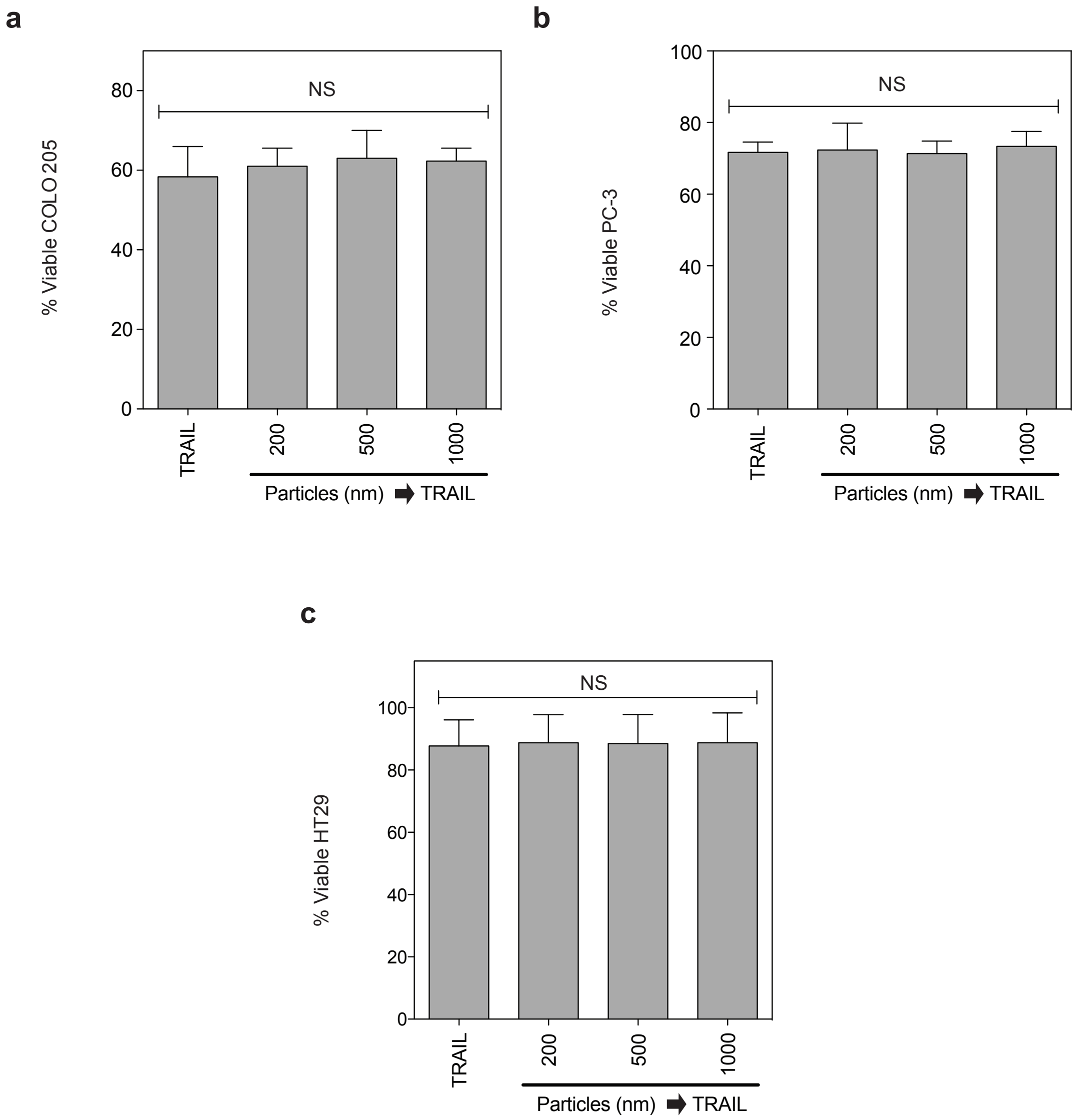

<u>Supplementary Figure 7:</u> TRAIL-mediated apoptosis is not amplified by polymeric particles under static conditions. (**a-c**) Colon COLO 205 (**a**), prostate PC-3 (**b**), and TRAIL-resistant colon HT29 (**c**) tumor cells were functionalized with polymeric polystyrene particles of varying sizes (200-1000 nm diameter) and treated with 0.1 μg mL^-1 TRAIL.). COLO 205, PC-3, and HT29 tumor cells were treated with TRAIL for 30 min, 1 h, and 6 h respectively. Tumor cells were then washed, incubated overnight, and assessed for cell viability using a CellTiter-Glo® luminescence assay. Data are reported as the mean ± s.e. Different treatment groups were compared for statistical significance using a one-way analysis of variance (ANOVA). NS: not significant. This finding suggests that a range of polymeric particle sizes conjugated to the tumor cell surface does not significantly alter TRAIL-mediated apoptosis under static conditions. Experiments were repeated 5 times.

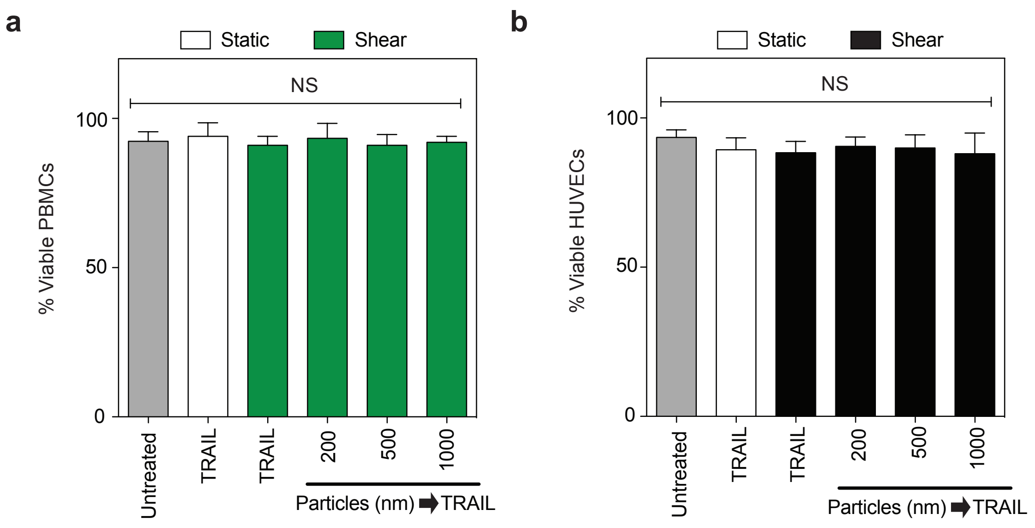

Supplementary Figure 8: TRAIL-mediated killing of normal cells is not amplified by polymeric particles in the presence of fluid shear stress. (**a,b**) Human peripheral blood mononuclear cells (PBMCs) (**a**) and human endothelial cell monolayers (**b**) were functionalized with polymeric polystyrene particles of varying sizes (200-1000 nm diameter) and treated with 0.1 µg mL^-1 TRAIL for 90 min under shear (2.0 dyn cm^-2) and static conditions. Cells were then washed, incubated overnight, and assessed for cell viability using a CellTiter-Glo® luminescence assay. Data are reported as the mean ± s.e. Different treatment groups were compared for statistical significance using a one-way analysis of variance (ANOVA) for multiple comparisons. NS: not significant. This finding suggests that a range of polymeric particle sizes conjugated to the surface of normal cells does not significantly alter TRAIL-mediated apoptosis under shear conditions. Experiments were repeated 5 times.

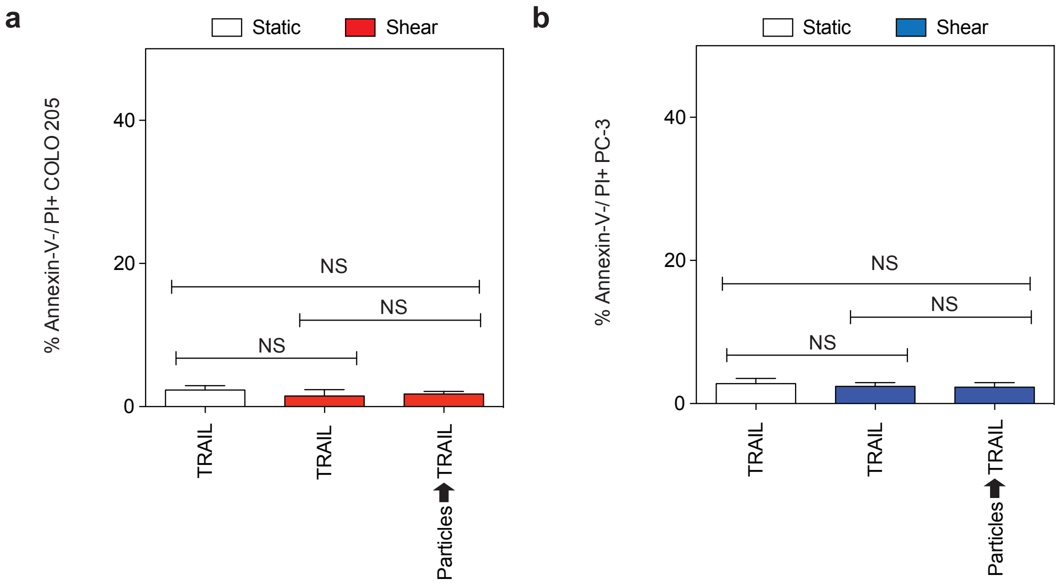

Supplementary Figure 9: Particle conjugation to the tumor cell surface does not affect cellular necrosis after treatment with TRAIL in the presence of a fluid shear force. (**a,b**) Colon COLO 205 (**a**) and prostate PC-3 (**b**) tumor cells were functionalized with polymeric polystyrene particles (500 nm diameter), treated with 0.1 µg mL^-1 TRAIL, and exposed to a fluid shear force (shear stress: 4.0 dyn cm^-2) for 30 min and 1 h, respectively. Tumor cells were then washed, placed in culture for 2 h, and

assessed for cellular necrosis using an annexin-V/propidium iodide (PI) flow cytometry assay. Annexin-V-/PI+ stained tumor cells were classified as necrotic. Data are reported as the mean ± s.e. Different treatment groups were compared for statistical significance using a Student's two-tailed t-test for two conditions and one-way analysis of variance (ANOVA) for multiple comparisons. NS: not significant. This finding suggests that polymeric particles conjugated to the tumor cell surface do not significantly affect cellular necrosis upon TRAIL treatment under a fluid shear force. Experiments were repeated 5 times.

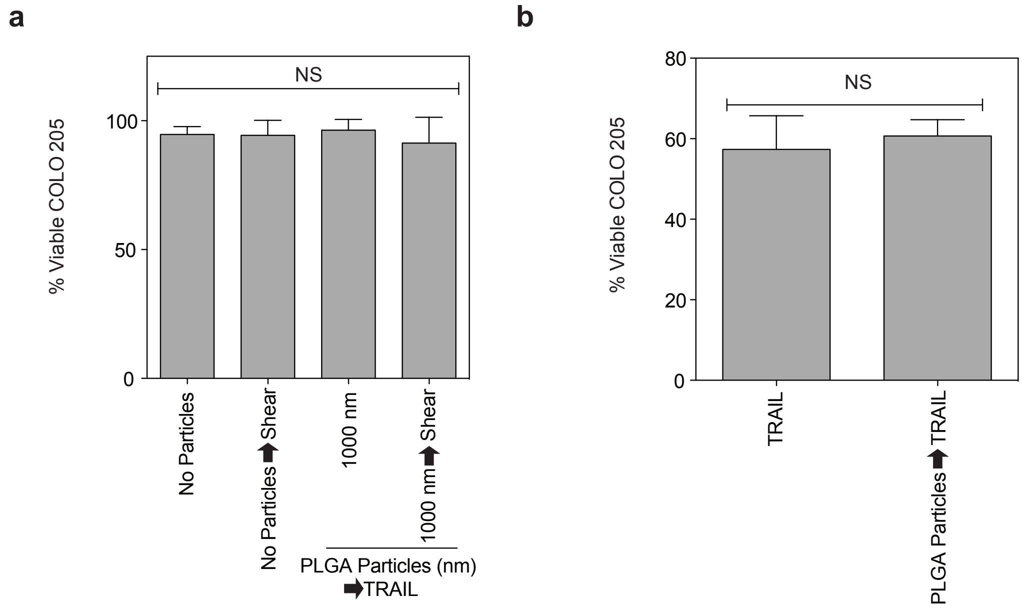

Supplementary Figure 10: Biodegradable PLGA particles do not affect tumor cell viability under fluid shear forces and do not affect TRAIL-mediated apoptosis under static conditions. (**a**) Colon COLO 205 tumor cells were functionalized with biodegradable PLGA particles (1000 nm diameter) and exposed to a fluid shear force (shear stress: 4.0 dyn cm^-2) for 30 min. Tumor cells were then washed, incubated overnight, and assessed for cell viability using a CellTiter-Glo® luminescence assay. NS: not significant. (**b**) COLO 205 cells were functionalized with biodegradable PLGA particles and treated with 0.1 µg mL^-1 TRAIL for 30 min. Tumor cells were then washed, incubated overnight, and assessed for cell viability using a CellTiter-Glo® luminescence assay. Data are reported as the mean ± s.e. Different treatment groups were compared for statistical significance using a Student's two-tailed t-test for two conditions and one-way analysis of variance (ANOVA) for multiple comparisons. NS: not significant. These data suggest that biodegradable PLGA particles conjugated to the tumor cell surface do not significantly affect cell viability under fluid shear force exposure and do not affect TRAIL-mediated apoptosis under static conditions. Experiments were repeated 3 times.

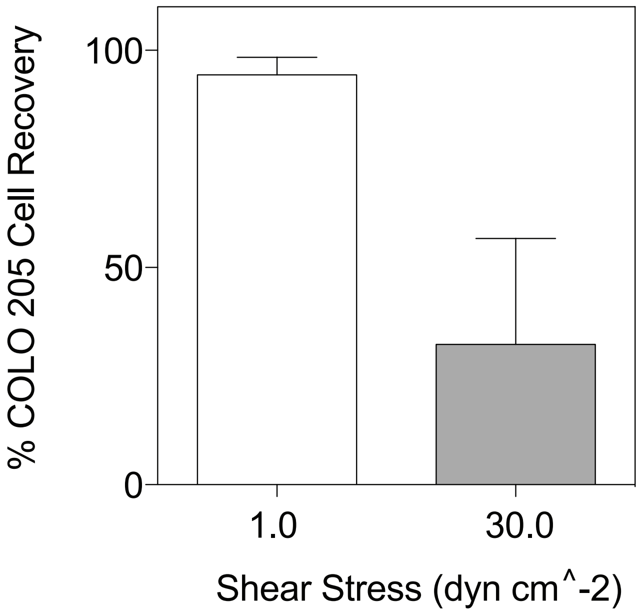

Supplementary Figure 11: High shear stress exposure in a cone-and-plate viscometer reduces cell recovery. COLO 205 tumor cells were exposed to low (1.0 dyn cm^-2) and high (30.0 dyn cm^-2) fluid shear stress values in a cone-and-plate viscometer for 30 min, removed from the device, and quantified. The percentage of cells recovered was normalized based on a non-sheared cell control sample. Data are reported as the mean ± s.e. These results indicate that tumor cells can easily be removed from the cone-and-plate viscometer and analyzed after low shear exposure, but can only be partially recovered after exposure to high shear stress. Experiments were repeated 3 times.

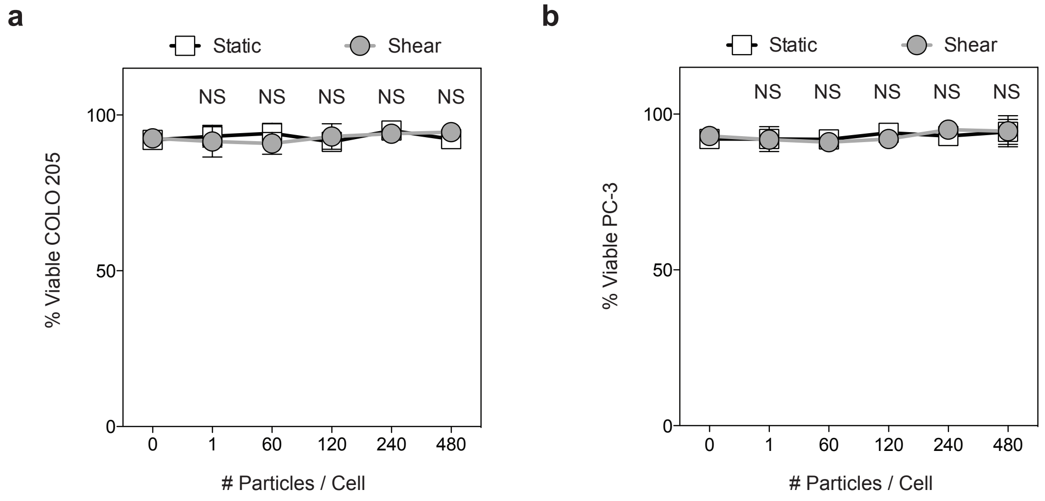

Supplementary Figure 12: Increased number of particles conjugated to tumor cell surface does not affect cell viability in the presence of a fluid shear force. (**a,b**) Viability of particle-functionalized COLO 205 (**a**) and PC-3 tumor cells (**b**) in the presence of a fluid shear force. Tumor cells were functionalized with 0-480 PS particles (500 nm diameter) per cell, and exposed to a fluid shear force (shear stress: 4.0 dyn cm^-2) or static conditions for 30 min and 1 h, respectively. Data are reported as the mean ± s.e. Different treatment groups were compared for statistical significance using a Student's two-tailed t-test. NS: not significant. These data suggest that the number of particles conjugated to the

tumor cell surface can be increased without affecting cell viability under shear and static conditions. Experiments were repeated 5 times.

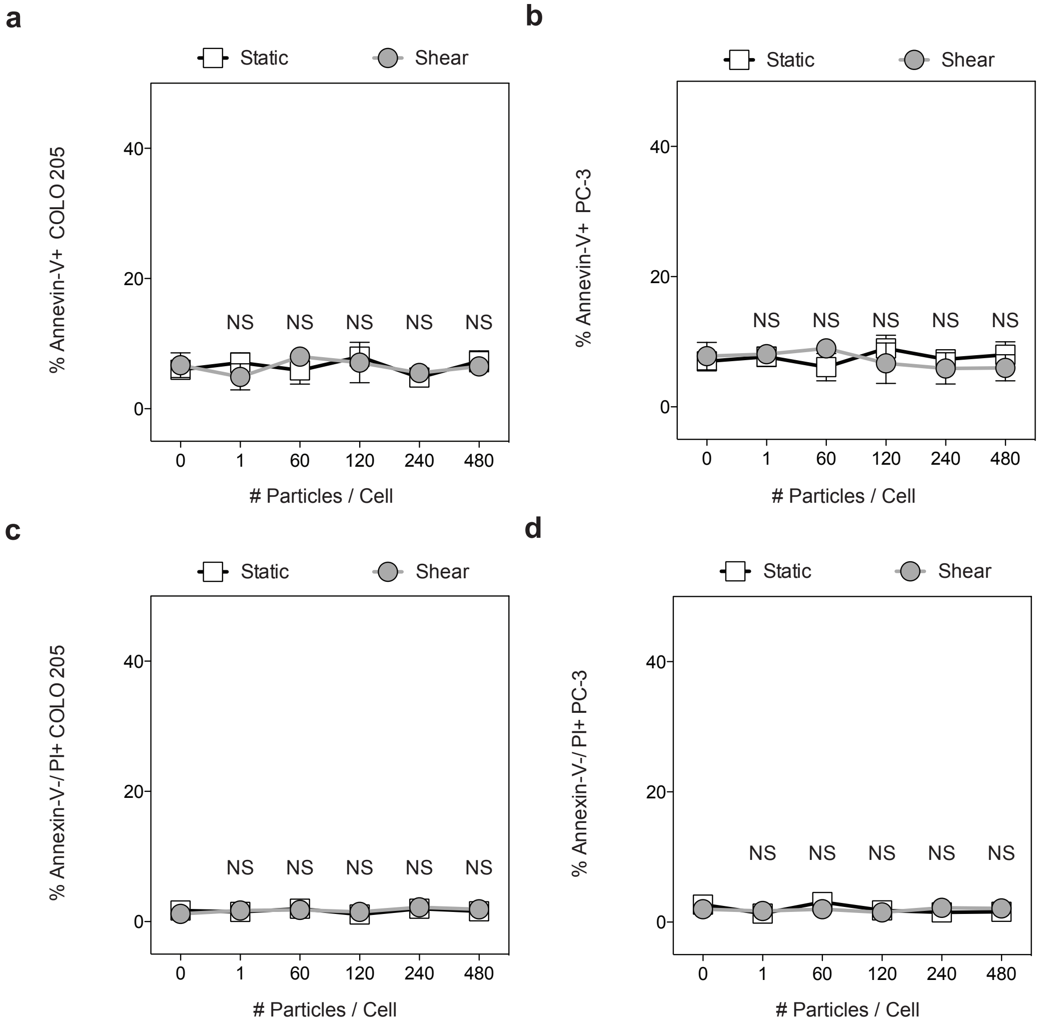

Supplementary Figure 13: Increased number of particles conjugated to tumor cell surface does not affect cellular apoptosis or necrosis in the presence of a fluid shear force. (**a,b**) Annexin-V labeling of particle-functionalized COLO 205 (**a**) and PC-3 tumor cells (**b**) in the presence of a fluid shear force. (**c,d**) Annexin-V-/propidium iodide (PI)+ labeling of particle-functionalized COLO 205 (**c**) and PC-3 tumor cells (**d**) in the presence of a fluid shear force. Tumor cells were functionalized with 0-480 PS particles (500 nm diameter) per cell, and exposed to a fluid shear force (shear stress: 4.0 dyn cm^-2) or static conditions for 30 min and 1 h, respectively. Tumor cells were then washed, incubated for 2 h, and stained using an annexin-V/PI flow cytometry assay. Cells positive for annexin-V were classified as apoptotic. Cells positive for PI and negative for annexin-V were classified as necrotic. Data are reported as the mean ± s.e. Different treatment groups were compared for statistical significance using a Student's two-tailed t-test. NS: not significant. These data suggest that the number of particles conjugated to the tumor cell surface can be increased without affecting cellular apoptosis and necrosis under shear and static conditions. Experiments were repeated 5 times.

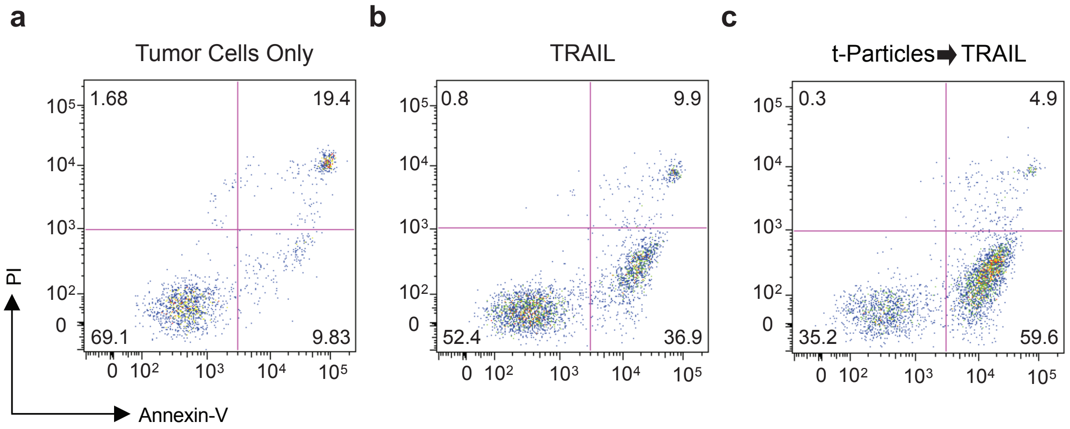

Supplementary Figure 14: Polymeric particles targeted to tumor cell surface *in vivo* amplify TRAIL-mediated apoptosis in the circulation. (**a-c**) Annexin-V/propidium iodide (PI) staining of tumor cells (**a**), tumor cells treated with TRAIL (**b**), and tumor cells treated with EpCAM-targeted PLGA particles *in vivo* followed by TRAIL therapeutic (**c**). Nu/nu mice were inoculated with GFP+ COLO 205 tumor cells via tail vein injection (2 x 10^6 cells), followed by injection with EpCAM-targeted PLGA particles (~500 particles per tumor cell injected). 30 mins post-particle injection, mice were treated with 100 µL of saline or soluble TRAIL therapeutic (sTRAIL; 1.5 µg mL^-1; TRAIL plasma concentration ~0.1 µg mL-1) via tail vein using a 30-guage needle. Blood (~200 µL) was collected via submandibular bleed, and circulating COLO 205 cells were separated from whole blood using Ficoll-Paque PLUS. After centrifugation, the buffy coat was collected, washed in buffer containing Ca^{2+}, and cultured for 4-6 h in multiwell plates in culture medium. Tumor cells were stained with an APC-conjugated Annexin-V apoptosis kit for flow cytometry analysis. Viable cells were identified as being negative for both Annexin-V and propidium iodide (PI), early apoptotic cells were positive for Annexin-V only, late apoptotic cells were positive for both Annexin-V and PI, and necrotic cells were positive for PI only. Five mice were used in each group.

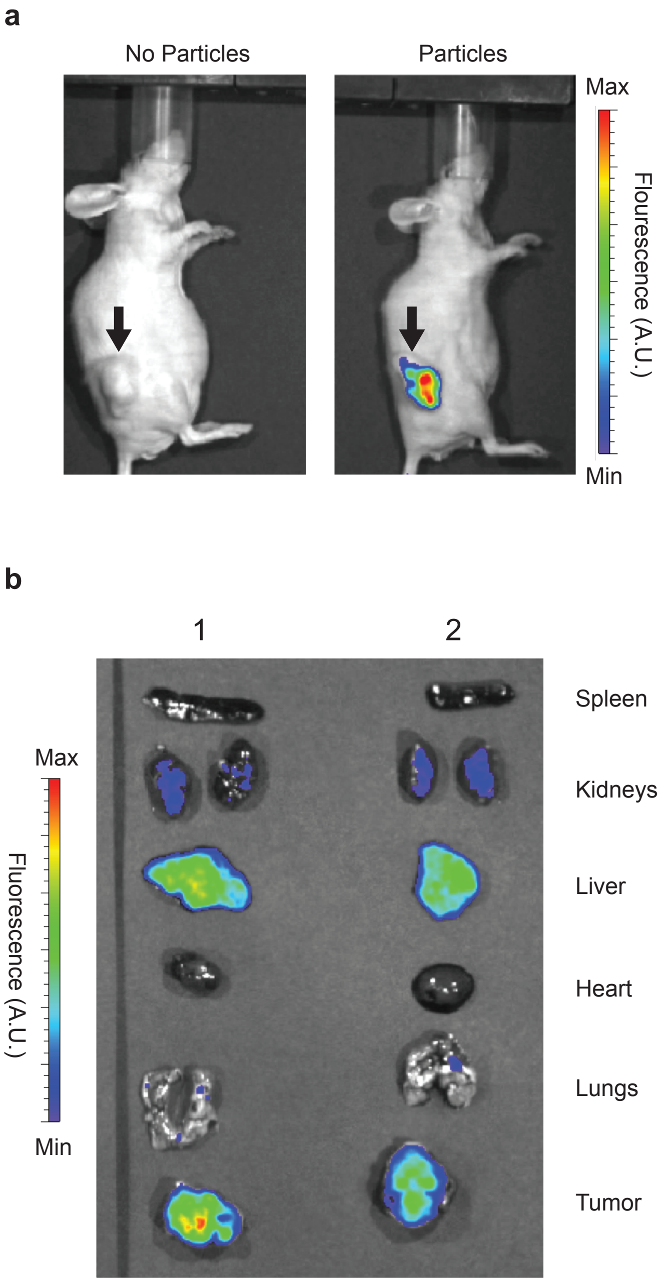

<u>Supplementary Figure 15:</u> Targeted polymeric particles localize within PC-3 tumor xenografts in nu/nu mice. (**a**) *In vivo* fluorescence imaging before and 3 h after fluorescent particle injection into nu/nu mice. Fluorescent biodegradable PLGA particles (size: 500 nm) functionalized with anti-epithelial cell adhesion molecule (EpCAM) antibodies were injected intravenously into nu/nu mice. (**b**) Fluorescence measurements of harvested organs 3 h post-injection of fluorescent anti-EpCAM PLGA particles. These results suggest that targeted particles localize within PC-3 xenografts 3 h post-injection.
